# Deep learning-based cross-attention fusion of multimodal MRI for survival prediction and risk stratification in IDH-wildtype glioblastoma: a multicenter study

**DOI:** 10.3389/fonc.2026.1867582

**Published:** 2026-08-24

**Authors:** Shichao Liu, Risheng Liang

**Affiliations:** 1 Department of Neurosurgery, Fujian Medical University Union Hospital, Fuzhou, Fujian, China; 2 Fujian Institute of Neurosurgery, Fuzhou, China

**Keywords:** cross-attention fusion, deep learning, glioblastoma, magnetic resonance imaging, multicenter study, risk stratification, survival prediction

## Abstract

**Background:**

Glioblastoma (GBM) exhibits profound molecular and spatial heterogeneity, complicating prognostic evaluations. While multiparametric MRI provides crucial multidimensional biological information, conventional end-to-end deep learning integration strategies, such as early or late fusion, often fail to capture complex nonlinear cross-modal interactions. We aimed to systematically evaluate a cross-attention fusion (CAF) architecture for GBM survival prediction and quantify its incremental prognostic value relative to existing clinical tools.

**Methods:**

In this multicenter retrospective study, 386 adults with IDH-wildtype, WHO grade 4 GBM were assembled from an institutional cohort (n = 226), the Chinese Glioma Genome Atlas (CGGA, n = 62), and The Cancer Genome Atlas (TCGA, n = 98). Using a unified 3D ResNet-18 backbone, we compared single-modality models, early fusion, late fusion, and CAF on preoperative T1-weighted, contrast-enhanced T1-weighted (T1CE), and T2-weighted MRI, and integrated the resulting deep learning risk score with routine clinical variables through multivariable Cox regression. Performance was assessed using Harrell’s C-index, time-dependent AUC, and decision curve analysis.

**Results:**

CAF showed numerically higher, more consistent C-index trends than early fusion, late fusion, and single-modality models (pooled C-index 0.629, 95% CI 0.594–0.664), although pairwise differences in time-dependent AUC were not statistically significant. Integrating clinical variables raised the pooled C-index to 0.691 (95% CI 0.660–0.721) in the treatment-era model, with comparable performance across the three cohorts (Local 0.688; CGGA 0.716; TCGA 0.689); a pre-treatment configuration excluding adjuvant therapy yielded a pooled C-index of 0.642. Under leave-one-cohort-out external validation, the combined model retained significant risk stratification in all held-out cohorts (C-index 0.63–0.71; all log-rank *P* < 0.01), albeit with attenuated discrimination. The deep learning risk score remained independent after multivariable adjustment (HR 1.41 per SD, 95% CI 1.26–1.57; *P* < 0.001). Kaplan–Meier analysis confirmed significant high- versus low-risk separation in all cohorts, and decision curve analysis showed greater net benefit than clinical-only and deep-learning-only models.

**Conclusion:**

The CAF-derived risk score offers prognostic information complementary to routine clinical variables, representing a promising noninvasive tool for individualized risk stratification when molecular profiling is incomplete or unavailable; these findings warrant prospective external validation before clinical use.

## Introduction

Glioblastoma (GBM) is the most common primary malignant brain tumor in adults. The 2021 World Health Organization (WHO) Classification of Tumors of the Central Nervous System explicitly defines GBM as an isocitrate dehydrogenase (IDH)-wildtype, WHO grade 4 diffuse astrocytic tumor (1). Although the current standard of care—maximal safe resection followed by concurrent chemoradiotherapy and adjuvant temozolomide—has been widely implemented in clinical practice (2), and various novel therapeutic strategies have entered clinical trials (3), the median overall survival remains approximately 15 months, with a five-year survival rate below 10% (4, 5). The substantial heterogeneity of GBM in molecular and imaging phenotypes complicates prognostic evaluations based on limited variables, hindering individualized treatment decisions and clinical trial stratification. Widely used prognostic tools, including clinical nomograms (6) and molecular markers such as O^6^-methylguanine-DNA methyltransferase (MGMT) promoter methylation (7, 8), exhibit limitations when evaluating cases with incomplete molecular profiles or strong spatial heterogeneity. These tools often rely on single-dimensional assessments and struggle to capture complex, nonlinear associations among tumor imaging features.

Multiparametric magnetic resonance imaging (MRI) provides important multidimensional biological information for the noninvasive evaluation of GBM, yet effectively extracting synergistic insights across modalities remains challenging. While end-to-end 3D deep learning has surpassed traditional radiomics by directly learning volumetric spatial contexts for survival prediction (9–13), conventional multimodal integration strategies—such as early or late fusion—often dilute modality-specific representations or fail to capture complex nonlinear cross-modal interactions (14). To address this, we introduce a cross-attention fusion (CAF) architecture that employs a multi-head attention mechanism within a deep feature space to explicitly model nonlinear dependencies among T1, T1CE, and T2 sequences. By utilizing single-modality features as query vectors to extract complementary information, CAF dynamically allocates modality weights at the individual patient level. This adaptive strategy aligns biologically with the marked inter- and intra-tumoral heterogeneity of GBM, dynamically prioritizing T1CE signals for blood-brain barrier disruption or T2 hyperintensities for occult infiltration (15, 16). Furthermore, the framework is deliberately restricted to these three routinely and universally acquired structural sequences—excluding sequences such as fluid-attenuated inversion recovery (FLAIR) or diffusion/perfusion-weighted imaging that are less consistently available across centers—to maximize applicability and reproducibility in multicenter settings.

Therefore, this study aims to systematically evaluate the CAF architecture for GBM survival prediction and quantify its incremental prognostic value relative to existing clinical tools. Utilizing a large multicenter retrospective cohort (n=386) across three independent institutions, we developed a unified deep learning framework to perform a head-to-head comparison of multimodal fusion strategies. Furthermore, we assessed the model’s cross-cohort transferability through both pooled cross-validation and a leave-one-cohort-out (LOCO) external validation, and evaluated the prognostic value of the deep learning risk score after adjusting for the available routine clinical and molecular variables. Collectively, this work provides a useful methodological reference for multimodal feature integration in neuro-oncology; in scenarios where molecular testing is restricted, routine MRI-based risk scores can serve as an objective, noninvasive supplementary dimension for individualized GBM risk stratification and clinical trial design.

## Materials and methods

### Study population and data sources

This is a multicenter retrospective study. Between January 2016 and December 2024, a total of 428 adult patients with GBM were evaluated at our institution. Inclusion criteria required histologically confirmed GBM with IDH-wildtype status, availability of complete preoperative T1-weighted images (T1WI, hereafter T1), contrast-enhanced T1-weighted images (T1CE), and T2-weighted images (T2WI, hereafter T2), and availability of overall survival (OS) data. To ensure diagnostic consistency, the IDH status of all early cases diagnosed before 2021 underwent strict re-evaluation according to the 2021 WHO Classification of Tumors of the Central Nervous System. Exclusion criteria included previous treatments, incomplete MRI sequences, severe motion artifacts, and IDH-mutant status. Based on these criteria, 202 patients were excluded from our institutional cohort (113 with no available preoperative MRI, 47 with no OS data, and 42 with non-IDH-wildtype status), resulting in a final inclusion of 226 cases.

**Figure 1:**
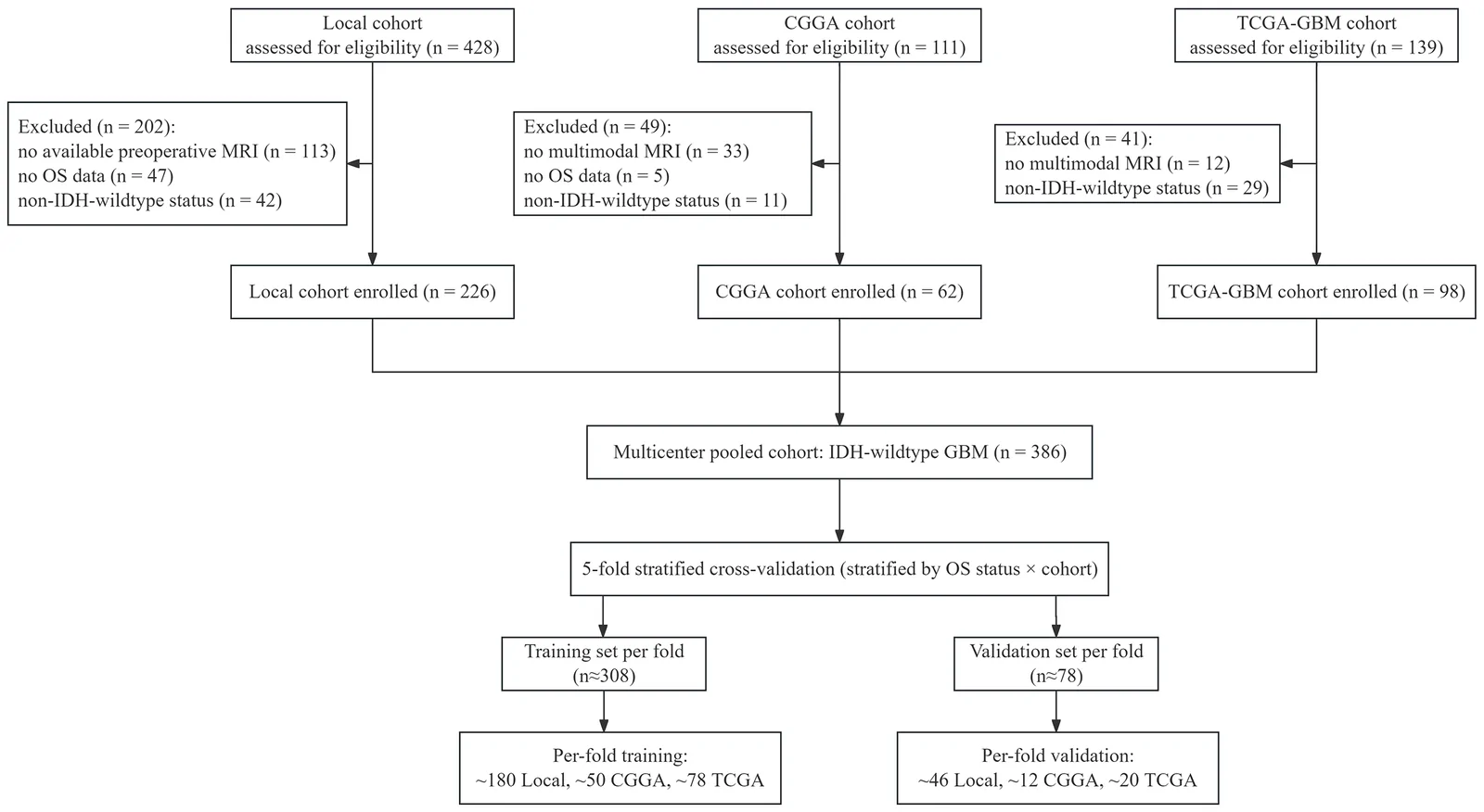
CONSORT-style flowchart of patient enrollment. An illustration of the inclusion and exclusion screening process for the three independent institutional cohorts. CGGA, Chinese Glioma Genome Atlas; GBM, glioblastoma; IDH, isocitrate dehydrogenase; MRI, magnetic resonance imaging; OS, overall survival; TCGA, The Cancer Genome Atlas.

For the two cohorts derived from public databases (CGGA, TCGA-GBM), the same inclusion and exclusion criteria as for the institutional cohort were applied. In the CGGA dataset, 111 patients were evaluated, and 49 were excluded (33 with no multimodal MRI, 5 with no OS data, and 11 with non-IDH-wildtype status), resulting in a final inclusion of 62 cases. In the TCGA-GBM dataset, 139 patients were evaluated, and 41 were excluded (12 with no multimodal MRI, and 29 with non-IDH-wildtype status), resulting in a final inclusion of 98 cases. Therefore, this study ultimately included a total of 386 patients from three independent institutions (**Figure 1**). For all three cohorts, OS was defined as the time from the date of initial surgical resection—which coincides with the date of histopathological diagnosis—to death from any cause, with patients alive at last contact censored at the date of last follow-up. This ensures a harmonized time origin across all three cohorts, as CGGA records OS from the date of primary surgery and TCGA-GBM from the date of initial pathological diagnosis. The study protocol was approved by the Institutional Review Board (Approval Number: 2026KY048), and given the retrospective design of this study, written informed consent was waived in accordance with the Institutional Review Board regulations. The use of the CGGA and TCGA public datasets complied with their respective data sharing agreements.

All 386 merged patients were evaluated using five-fold stratified cross-validation. The stratification strategy was implemented based on a joint distribution matrix of cohort origin (Local/CGGA/TCGA) and outcome event status (censored vs. dead) to ensure that each fold maintained consistency with the overall population in terms of center origin and event rate. Within each fold, approximately 308 cases were used for training and 78 for validation. In addition to reporting the overall pooled C-index, model performance was also reported separately by cohort (Local, CGGA, TCGA) to evaluate the cross-center generalization capability of the models.

### MRI acquisition and preprocessing

**Figure 2:**
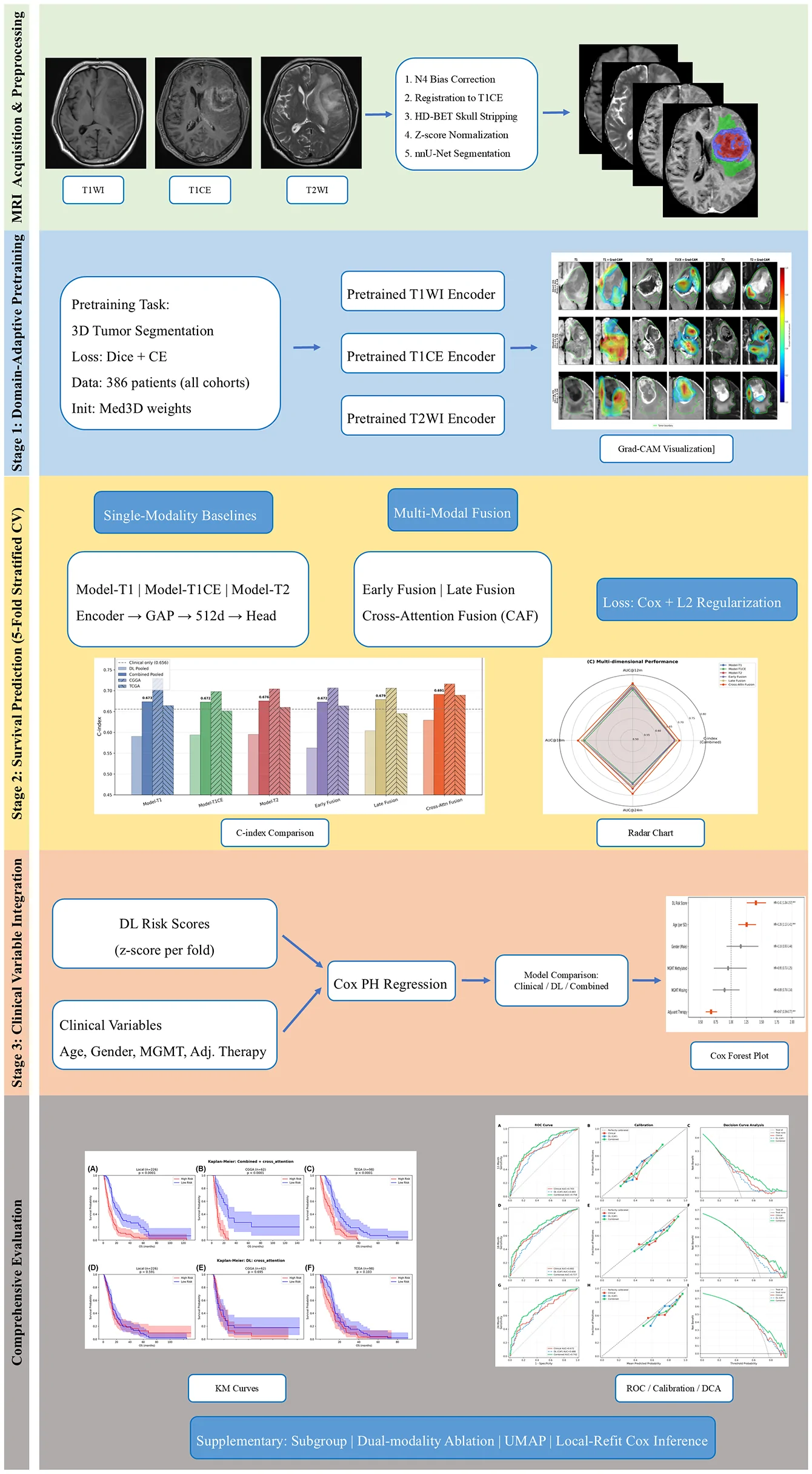
Study workflow. (1) Acquisition of T1, T1CE, and T2 MRI plus clinical data for 386 patients; (2) preprocessing (N4 bias-field correction, ANTs registration to T1CE and the SRI24 atlas, skull stripping, intensity normalization, nnU-Net tumor segmentation, and 128 × 128 × 128 ROI cropping); (3) two-stage deep learning (segmentation-supervised domain-adaptive pretraining, then survival fine-tuning with the Cox partial-likelihood loss); and (4) evaluation by five-fold stratified cross-validation. ANTs, Advanced Normalization Tools; nnU-Net, no-new-Net; ROI, region of interest.

An overview of the workflow of this study is illustrated in **Figure 2**. MRI examinations for our institutional cohort were performed on 1.5–3.0 T scanners (Siemens, Philips, GE) using 8–64 channel head coils. The scanning protocol included 3D volumetric T1-weighted sequences (repetition time [TR] = 8.4–1600 ms; echo time [TE] = 1.9–11 ms; field of view [FOV] = 220 × 220 to 256 × 256 mm; slice thickness = 1 mm and 5 mm; matrix = 240 × 240 to 256 × 248), acquired before and after intravenous injection of gadolinium contrast agent (gadoterate meglumine, 0.1 mmol/kg). The parameters for the post-contrast sequences were identical, with the slice thickness fixed at 1 mm. The parameters for the T2-weighted sequences were TR/TE = 4850–5100 ms/89–131 ms (FOV = 185 × 220 to 220 × 220 mm; slice thickness = 5 mm; matrix = 448 × 256 to 640 × 384). The CGGA dataset was obtained from the Chinese Glioma Genome Atlas (17), which provided skull-stripped images (T1_bet, CE_bet, T2_bet). The TCGA-GBM dataset was sourced from the Brain Tumor Segmentation (BraTS) 2021 challenge (18) and had undergone standardized preprocessing, including registration, skull stripping, and 1 mm isotropic resampling.

The preprocessing pipeline for our institutional cohort was based on the brainles-preprocessing toolkit (Atlas-Centric mode). This involved N4 bias field correction for all modalities (19), linear co-registration of T1 and T2 to the T1CE space using Advanced Normalization Tools (ANTs), with T1CE as the reference modality (20), registration to the BraTS SRI24 standard atlas space, skull stripping, and percentile-based intensity normalization mapping the 0.1–99.9 percentile to the [0, 1] interval. Tumor segmentation was performed using the no-new-Net (nnU-Net) models trained on the BraTS-2023 adult-glioma dataset (21, 22): a four-sequence model (T1, T1CE, T2, FLAIR) for the Local and TCGA cohorts, and a three-sequence model (T1, T1CE, T2) for CGGA, which lacks FLAIR. These models’ segmentation accuracy, assessed by 5-fold cross-validation against expert ground truth on the BraTS-2023 dataset, yielded whole-tumor Dice coefficients of 0.938 and 0.910, and 95% Hausdorff distances of 3.56 and 4.64 mm for the four- and three-sequence models, respectively (**Supplementary Table 1**). Because expert tumor annotations were unavailable for the study cohorts, both registration and automated segmentation quality were instead systematically assessed by a neuroradiologist with 13 years of neuroimaging experience via visual inspection in a single ITK-SNAP session. For registration, using linked cross-hair navigation and image-overlay/contour inspection, the reviewer verified intra-subject alignment (T1 and T2 images to the T1CE reference) and spatial normalization to the SRI24 atlas, focusing on key anatomical landmarks, including ventricular margins, the interhemispheric midline, and cortical/skull-base boundaries. Simultaneously, the automated segmentations for all three cohorts were evaluated. Ultimately, no cases exhibited gross misregistration or segmentation failures, no manual re-editing was applied, and none required exclusion or re-registration. The region of interest (ROI) was defined as the minimum bounding box of the whole-tumor mask (the union of all tumor labels: necrotic/non-enhancing core, peritumoral edema, and enhancing tumor), expanded by a 10-voxel (approximately 10 mm at the 1 mm isotropic processing resolution) margin symmetrically to retain surrounding peritumoral parenchyma beyond the segmented whole-tumor region, where GBM is known to infiltrate microscopically, and resampled to a standardized volume of 128 × 128 × 128 voxels. The tumor mask was used solely to define this field of view and was not provided as an input channel to the survival model.

### Deep learning model architecture

**Figure 3:**
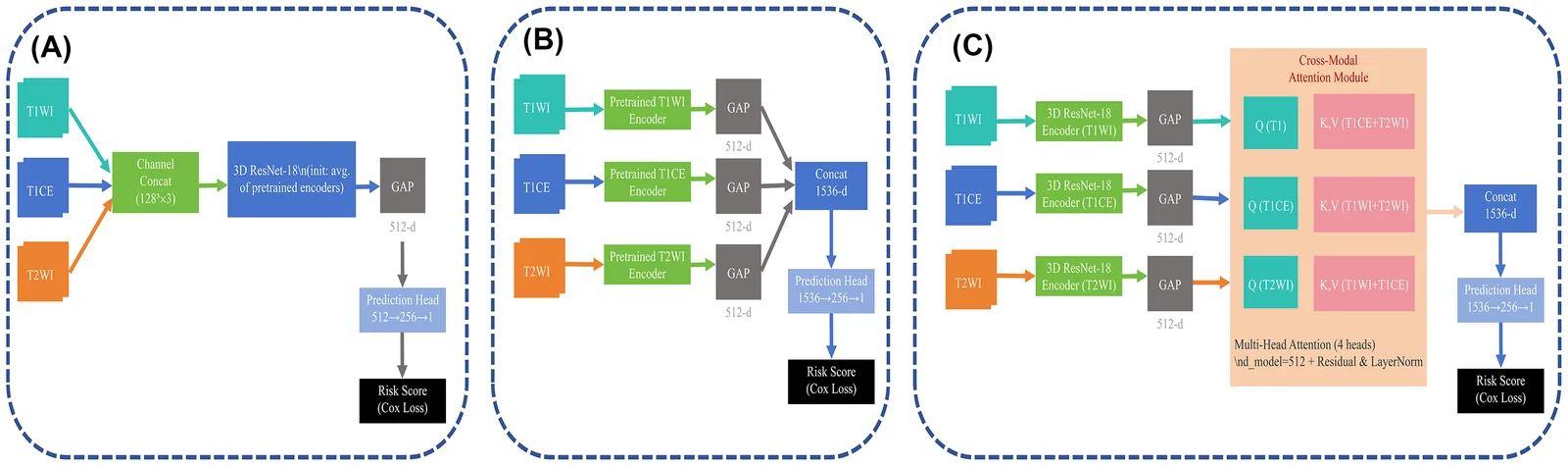
Deep learning model architectures. All six models share a unified 3D ResNet-18 backbone. **(A)** Early Fusion (EF): T1/T1CE/T2 concatenated as a 3-channel input to a single encoder. **(B)** Late Fusion (LF): three independent encoders produce 512-D features that are concatenated (1536-D) and passed to a survival head. **(C)** Cross-Attention Fusion (CAF): three encoders followed by a multi-head cross-attention module (4 heads, d_model = 512) in which each modality queries the other two, yielding a refined 1536-D representation. Single-modality baselines (Model-T1/T1CE/T2) apply the same backbone to one sequence. CAF, cross-attention fusion; EF, early fusion; LF, late fusion.

This study systematically compared six models, comprising three single-modality baseline models (Model-T1, Model-T1CE, Model-T2) and three multimodal fusion architectures (early fusion, late fusion, cross-attention fusion), all sharing the identical 3D ResNet-18 backbone network (23) (**Figure 3**). We adopted the 3D ResNet-18 as the unified backbone to balance feature-extraction capacity against overfitting risk at the present sample size (n = 386) and to leverage publicly available 3D medical pretrained weights (Med3D) within our two-stage paradigm. Importantly, a relatively lightweight, shared backbone ensures that performance differences are attributable to the fusion design rather than to backbone capacity, consistent with the study’s head-to-head aim. A detailed comparison of parameter counts and memory considerations across ResNet depths is provided in the **Supplementary Material** (**Supplementary Table 2**). The three single-modality baseline models process a single MRI modality individually, extract features using the pretrained 3D ResNet-18 encoder, generate a 512-dimensional feature vector via global average pooling, and then output through a fully connected survival prediction head (512 → 256 → 1), optimized using the Cox partial likelihood loss.

### Two-stage training strategy

To mitigate the challenge of limited annotated samples in survival prediction tasks, a two-stage training paradigm was adopted in this study. The first stage involved domain-adaptive pretraining, where three independent 3D ResNet-18 encoders (one for each modality, with no weight sharing among encoders and no cross-modal information interaction during training) underwent 3D tumor segmentation pretraining on all 386 patients. This pretraining was global—performed once on the full dataset before cross-validation—and used only tumor-segmentation supervision (Dice + cross-entropy loss against tumor masks); no overall-survival times or event labels were used at any point during Stage 1. The resulting encoder weights were subsequently loaded identically into every cross-validation fold; because validation-fold images (though not their outcomes) contributed to this representation learning, we additionally performed a fold-specific pretraining sensitivity analysis to assess whether this introduced optimistic bias in discrimination. The network structure included encoders initialized with Med3D pretrained weights and a lightweight U-Net style decoder. Training used a combination of Dice loss and cross-entropy loss (1:1 weight), an AdamW optimizer (learning rate = 1 × 10^-^³, weight decay = 1 × 10^-4^), and a cosine annealing schedule for 100 epochs with a batch size of 2. Data augmentation included random flipping, rotation (± 15°), and intensity shift (± 10%). This stage generated three sets of domain-adapted encoder weights corresponding to the T1, T1CE, and T2 modalities.

The second stage was survival prediction fine-tuning. Following the loading of the first-stage pretrained encoder weights, the decoders were discarded, and a differential learning rate strategy was employed. The backbone network stem and layers 1–3 were completely frozen, layer 4 was fine-tuned with a lower learning rate (5 × 10^-5^), and the fusion modules and prediction heads were trained with a base learning rate (1 × 10^-4^). The loss function was the Cox partial likelihood loss combined with an L2 risk score regularization (λ = 0.01). Because this loss was evaluated within each mini-batch, the risk set for each event was restricted to the samples present in that batch; it therefore constitutes a stochastic mini-batch approximation of the full-cohort Cox partial likelihood rather than its exact form. Training used an AdamW optimizer (weight decay = 1 × 10^-^³), followed by a 10-epoch warmup and a cosine annealing schedule for a maximum of 100 epochs, utilizing an early stopping strategy (patience = 30, based on the validation set C-index), a batch size of 4, and a Dropout of 0.5. All six models started from the exact same first-stage weights to ensure that any performance differences were entirely attributable to the fusion architecture design. Under this policy (Stage-1 stem and layers 1–3 frozen; layer 4, fusion modules, and the survival head trainable), the total and trainable parameter counts were 33.29 M and 25.04 M for each single-modality model (Model-T1, Model-T1CE, and Model-T2, which are architecturally identical), 33.34 M and 25.04 M for early fusion (EF), 99.87 M and 75.12 M for late fusion (LF), and 106.18 M and 81.43 M for CAF. The detailed per-model counts are tabulated in **Supplementary Table 2**.

### Fusion architectures

EF concatenates the T1, T1CE, and T2 volumes along the channel dimension into a three-channel input (128 × 128 × 128 × 3), and the first convolutional layer kernel is expanded for the three-channel input. Its initialization adopts a hybrid strategy where the stem layer concatenates the stem weights of the three single-modality first-stage models along the input channel dimension, allowing each input channel to inherit the feature response patterns of its corresponding modality after domain adaptation. The deep ResNet layers (layer1–layer4) are initialized with the element-wise average of the three single-modality encoder weights. Of note, averaging the deep layers provides a reasonable starting point for the joint three-modality input, thereby ensuring consistent pretraining information in cross-architecture comparisons (all models start from the same set of first-stage weights). After initialization, it is processed by a single 3D ResNet-18 encoder and output via global average pooling and a survival prediction head.

LF employs three independent encoders (each loaded with the first-stage weights of the corresponding modality) to extract 512-dimensional features from T1, T1CE, and T2 individually, which are then concatenated into a 1536-dimensional representation and output through a fully connected survival prediction head (1536 → 256 → 1).

CAF similarly employs three independent encoders to extract modality-specific features, subsequently applying a cross-modal attention module. The feature map of each modality (from the layer 4 output, flattened into a sequence) serves as the Query, while the concatenated features of the other two modalities act as Key and Value. Through a multi-head cross-attention mechanism (4 heads, d_model = 512, Dropout = 0.1) combined with residual connections and layer normalization, three attention-refined 512-dimensional features are generated, concatenated (1536 dimensions), and output through the survival prediction head. Because LF and CAF share an identical three-encoder backbone and 1536-dimensional concatenated representation feeding the prediction head, differing only in the cross-attention module (which contributes approximately 6% of the total parameter count; **Supplementary Table 2**), the two architectures are directly comparable in terms of backbone capacity.

### Clinical variable integration

The risk scores from the respective deep learning models and clinical variables were incorporated into a Cox proportional hazards regression model (lifelines CoxPHFitter, L2 penalty coefficient = 0.1) for joint modeling. To eliminate scale differences in the deep learning risk scores and approximate an independent inference scenario, the risk scores for the validation set in each fold were strictly standardized using the mean and standard deviation calculated exclusively from the training set of that fold using z-score normalization (dl_risk_std = (score − μ_train)/σ_train). Similarly, the Cox coefficient estimation was independently fitted within the scope of the training set, thereby preventing data leakage across folds or validation stages, allowing for a systematic evaluation of different fusion architectures after clinical integration. The included clinical features consisted of age (standardized), gender, MGMT promoter methylation status, and adjuvant therapy status. Of these, the imaging-derived risk score, age, and sex are derived from preoperative data; MGMT methylation status is typically obtained peri-operatively from resected or biopsied tumor tissue and is generally available before treatment initiation; adjuvant therapy status, however, becomes known only after surgery and treatment planning is finalized. Accordingly, the full combined model represents a treatment-era prognostic model rather than a strictly preoperative tool; to characterize a configuration restricted to pre-treatment information, we additionally fitted a model that excluded adjuvant therapy. Extent of resection (EOR) and Karnofsky Performance Status (KPS), although well-established prognostic factors in GBM, were not systematically recorded in the public cohorts and were therefore not incorporated into the primary multivariable model; their potential influence was instead examined in a dedicated sensitivity analysis restricted to the institutional cohort. Considering the high rate of missing MGMT testing in routine clinical practice (46.6%) and the principle that patients lacking this test should not be excluded from model inputs in clinical deployment scenarios, this study treated missing MGMT values as an independent “Unknown” status, using mode imputation for the base variable to satisfy the numerical input requirements of the Cox model, and simultaneously introduced a binary missing indicator variable to explicitly preserve any prognostic information potentially carried by the missing pattern (missing not at random, MNAR). The applicability and methodological validity of this missing-indicator method in clinical prognostic research have been systematically explored in the statistical literature (24). This phase maintained the same five-fold cross-validation split as the second stage, comparing three configurations: clinical-variables-only, deep-learning-only, and a combined model (deep learning + clinical variables).

### Statistical analysis and validation strategy

The primary endpoint was overall survival. The primary evaluation metric was Harrell’s concordance index (C-index) (25), reported as the mean ± standard deviation across folds and the overall pooled value. Secondary evaluation metrics included time-dependent area under the receiver operating characteristic curve (AUC) at 12, 18, and 24 months, calibration curves, and decision curve analysis (DCA) (26). For risk stratification, patients were divided into high- and low-risk groups using a median risk score cutoff, and intergroup differences were compared using Kaplan-Meier survival curves and log-rank tests. For the pooled cross-validation analyses, the cutoff was the cohort-specific median of the out-of-fold (cross-validated) risk scores within each cohort. For the locally-fitted external-inference analysis and the LOCO external validation, a single prespecified cutoff—the median risk score of the training cohort(s)—was fixed on the training data and applied unchanged to the external cohort(s), consistent with intended clinical use. Multivariable Cox proportional hazards regression was used to evaluate the independent prognostic value of the deep learning risk score after adjusting for clinical covariates, reporting hazard ratios (HR) and 95% confidence intervals (CI). The proportional-hazards assumption was assessed for every covariate using scaled Schoenfeld residuals (global and per-covariate tests, under both rank and Kaplan–Meier time transforms); where non-proportionality was detected, a Cox model stratified by cohort (allowing a separate baseline hazard per data source) was fit as a confirmatory sensitivity analysis. Differences in time-dependent AUCs between models were compared using DeLong’s test (27).

In addition to the cohort-stratified Cox model fitted as a confirmatory sensitivity analysis for the proportional-hazards assumption, we performed several further sensitivity analyses to assess the robustness of the deep learning risk score and the main model. To assess the robustness of the deep learning risk score’s prognostic value to key covariates available only in the institutional cohort, we performed a sensitivity analysis in that cohort, refitting the multivariable Cox model with additional adjustment for EOR (gross-total vs. non-gross-total resection) and KPS (≥ 70 vs. < 70), and evaluating their incremental contribution using a likelihood-ratio test. Additionally, to evaluate a pre-treatment configuration, we refitted the multivariable model after excluding adjuvant therapy (a post-treatment-planning variable), retaining covariates available before treatment initiation (age, sex, and MGMT status with its missing indicator) alongside the imaging-derived risk score, and compared discrimination with and without adjuvant therapy using the identical out-of-fold cross-validation scheme. Furthermore, to assess the robustness of results to the MGMT missing-data handling strategy, we performed a complete-case sensitivity analysis restricted to the 206 patients with documented MGMT methylation status, entering MGMT directly as a binary variable without imputation or a missing indicator; the representativeness of this subset was assessed by comparing patients with and without known MGMT status across baseline and survival-relevant characteristics using Welch’s t-test and the χ² test. As an exploratory sensitivity analysis of sequence completeness, we evaluated the effect of adding FLAIR within the subcohort in which FLAIR was available (Local and TCGA; n = 324; CGGA lacks FLAIR). Two CAF models were trained and evaluated under identical stratified 5-fold cross-validation splits: a three-sequence model (T1, T1CE, T2; identical to the primary model) and a four-sequence model incorporating an additional FLAIR encoder and its corresponding cross-attention stream. Because no segmentation-pretrained FLAIR encoder was available, the FLAIR stream was initialized using the pretrained T2 encoder weights. Discrimination was summarized by the out-of-fold C-index and time-dependent AUC, and the between-model difference in C-index was assessed via a 1, 000-resample paired bootstrap. This analysis was strictly exploratory and was not used to select the primary model.

To complement the pooled cross-validation with an approximate external-validation design, we additionally performed a LOCO analysis. In turn, each of the three cohorts was entirely withheld as an untouched external test set, while the deep-learning survival model (Stage-2 fine-tuning), the per-fold z-score normalization statistics, and the downstream Cox proportional hazards layer were trained exclusively on the remaining two cohorts. Consequently, the held-out cohort contributed no samples to survival-model training or threshold selection. Stage-1 domain-adaptive pretraining—which uses tumor-segmentation supervision only, with no survival outcomes—was retained on the full cohort, as in the main pipeline; the influence of this choice on discrimination was evaluated separately by the fold-specific pretraining sensitivity analysis. External discrimination was quantified using the C-index and time-dependent AUC, and binary risk stratification was assessed via Kaplan–Meier analysis using a single prespecified cutoff—the median risk score of the held-in (training) cohorts—that was fixed on the training data and applied unchanged to the held-out cohort, consistent with intended clinical use.

Furthermore, exploratory subgroup analyses were performed within clinically relevant sub-populations (age ≥ 60 years vs. < 60 years, male vs. female, MGMT methylated vs. unmethylated, adjuvant therapy vs. no adjuvant therapy), with 95% confidence intervals obtained by nonparametric bootstrap resampling (1, 000 iterations; percentile method; fixed random seed for reproducibility). Resampling was conducted at the patient level, drawing cases with replacement up to the original sample size, and was applied directly to the fixed, pooled out-of-fold (cross-validated) predictions; fold assignments were not resampled. Resampling was not stratified by event status; any resample containing insufficient events (fewer than 2 for the C-index; fewer than 3 for the time-dependent AUC) was discarded and redrawn. For cohort-specific and subgroup-specific intervals, resampling was conditioned within the relevant cohort or subgroup. This procedure was applied uniformly to the C-index, time-dependent AUC, integrated Brier score, and calibration metrics; for the time-dependent AUC and integrated Brier score, inverse-probability-of-censoring weights were derived from the censoring distribution of the reference cohort. These analyses were hypothesis-generating; several subgroups were small, and no correction for multiple comparisons was applied. Calibration was assessed at 12, 18, and 24 months both graphically (calibration curves) and quantitatively by the observed/expected (O/E) ratio, the calibration slope, and the calibration-in-the-large (calibration intercept). The slope and intercept were robustly estimated from jackknife pseudo-observations of the event probability utilizing a complementary log-log link function. As an exploratory analysis of potential clinical utility, DCA was used to evaluate the net benefit of using the model to flag high-risk patients for an intensified-management pathway (more frequent neuro-imaging surveillance, earlier consideration of salvage therapy, or clinical-trial prioritization) across a range of threshold probabilities corresponding to the predicted probability of death by the respective time point. All statistical analyses were performed using Python 3.11 (lifelines 0.27, scikit-survival 0.22, scikit-learn 1.3), with a two-sided *P* < 0.05 considered statistically significant. A complete list of hyperparameters for the three stages is provided in **Supplementary Table 3**. To ensure reproducibility, all experiments used a fixed global random seed (42), rendering the stratified five-fold splits fully regenerable, with the complete software and hardware environment and detailed end-to-end pseudocode of the pipeline (including the Cox partial-likelihood loss) provided in the **Supplementary Material** (**Supplementary Algorithm S1**).

## Results

### Baseline characteristics of patients

A total of 386 IDH-wildtype GBM patients were included (**Table 1**; **Figure 1**). The mean ages of the institutional, CGGA, and TCGA cohorts were 57.0 ± 12.6 years, 51.4 ± 14.0 years, and 59.0 ± 13.9 years, respectively (*P* = 0.002), with similar male-to-female ratios (males comprising 56.5%–62.8%, *P* = 0.652). MGMT promoter methylation status was available for 206 of the 386 cases (53.4%), and the methylation rates were similar across cohorts (39.6%–45.5%, *P* = 0.777). Significant differences existed in the adjuvant therapy rates, with the highest in the CGGA cohort (93.5%) and the lowest in our institutional cohort (69.0%) (*P* < 0.001). The median OS was 12.4 months (interquartile range [IQR]: 6.9–19.5) in the Local cohort, 12.3 months (IQR: 8.3–25.5) in CGGA, and 12.6 months (IQR: 6.8–20.8) in TCGA, showing no statistically significant differences across the three cohorts (*P* = 0.441). The event rates were 74.8%, 87.1%, and 91.8%, respectively.

**Table 1:** Baseline clinicopathological characteristics of the study population.

Characteristics	Internal cohort (n=226)	CGGA (n=62)	TCGA-GBM (n=98)	Total (n=386)	P-value
Age, mean ± SD	57.0 ± 12.6	51.4 ± 14.0	59.0 ± 13.9	56.6 ± 13.4	0.002
Age, median (IQR)	59 (49–67)	54 (45-61)	60 (53-69)	58 (50-66)	
Gender, Male n (%)	142 (62.8%)	35 (56.5%)	61 (62.2%)	238 (61.7%)	0.652
MGMT methylated, n (%)	46/101 (45.5%)	21/53 (39.6%)	23/52 (44.2%)	90/206 (43.7%)	0.777
MGMT missing, n (%)	125 (55.3%)	9 (14.5%)	46 (46.9%)	180 (46.6%)	
Adjuvant therapy, n (%)	156/226 (69.0%)	58/62 (93.5%)	84/98 (85.7%)	298/386 (77.2%)	<0.001
OS, median months (IQR)	12.4 (6.9-19.5)	12.3 (8.3-25.5)	12.6 (6.8-20.8)	12.6 (7.0-20.7)	0.441
Death event, n (%)	169 (74.8%)	54 (87.1%)	90 (91.8%)	313 (81.1%)	

IQR, interquartile range; MGMT, O^6^-methylguanine-DNA methyltransferase; OS, overall survival; SD, standard deviation. P-values were calculated using Analysis of Variance (ANOVA) for continuous variables and Chi-square or Fisher’s exact test for categorical variables.

### Discriminative performance of deep learning models

The predictive performances of the three single-modality deep learning models were comparable (**Table 2**). Model-T1CE achieved the highest average validation C-index (0.600 ± 0.016), followed by Model-T2 (0.598 ± 0.019) and Model-T1 (0.593 ± 0.032), with pooled C-indices of 0.595 for T2, 0.594 for T1CE, and 0.590 for T1, showing no significant differences among them. Model-T1 exhibited the highest C-index in the CGGA and TCGA cohorts (CGGA: 0.622; TCGA: 0.606), while Model-T1CE attained the highest C-index in the institutional cohort (0.601).

**Table 2:** Performance comparison of single-modality models and multimodal fusion architectures.

Model	Modality/architecture	Cross-validation C-index (Mean ± SD)	Pooled C-index (95% CI)	Internal cohort C-index (95% CI)	CGGA C-index (95% CI)	TCGA C-index (95% CI)
Clinical Only	T1	0.654 ± 0.034	0.656 (0.624-0.688)	0.654 (0.608-0.696)	0.695 (0.606-0.764)	0.647 (0.571-0.709)
DL-only (T1)	T1	0.593 ± 0.032	0.590 (0.556-0.623)	0.572 (0.523-0.625)	0.622 (0.530-0.698)	0.606 (0.542-0.669)
Combined (T1)	T1	0.674 ± 0.030	0.673 (0.643-0.706)	0.666 (0.621-0.710)	0.730 (0.659-0.792)	0.664 (0.593-0.728)
Clinical Only	T1CE	0.654 ± 0.034	0.656 (0.624-0.688)	0.654 (0.608-0.696)	0.695 (0.606-0.764)	0.647 (0.571-0.709)
DL-only (T1CE)	T1CE	0.600 ± 0.016	0.594 (0.558-0.631)	0.601 (0.555-0.653)	0.576 (0.488-0.656)	0.583 (0.513-0.645)
Combined (T1CE)	T1CE	0.673 ± 0.028	0.672 (0.637-0.706)	0.678 (0.633-0.720)	0.698 (0.621-0.766)	0.651 (0.568-0.714)
Clinical Only	T2	0.654 ± 0.034	0.656 (0.624-0.688)	0.654 (0.608-0.696)	0.695 (0.606-0.764)	0.647 (0.571-0.709)
DL-only (T2)	T2	0.598 ± 0.019	0.595 (0.559-0.631)	0.582 (0.532-0.628)	0.622 (0.549-0.687)	0.601 (0.532-0.668)
Combined (T2)	T2	0.682 ± 0.027	0.676 (0.643-0.706)	0.677 (0.630-0.717)	0.704 (0.630-0.774)	0.660 (0.586-0.720)
Clinical Only	T1 + T1CE + T2	0.654 ± 0.034	0.656 (0.624-0.688)	0.654 (0.608-0.696)	0.695 (0.606-0.764)	0.647 (0.571-0.709)
DL-only (Early Fusion)	T1 + T1CE + T2	0.570 ± 0.032	0.563 (0.528-0.600)	0.560 (0.505-0.610)	0.556 (0.467-0.644)	0.595 (0.524-0.665)
Combined (Early Fusion)	T1 + T1CE + T2	0.669 ± 0.039	0.672 (0.641-0.705)	0.668 (0.622-0.710)	0.706 (0.624-0.781)	0.663 (0.589-0.726)
Clinical Only	T1 + T1CE + T2	0.654 ± 0.034	0.656 (0.624-0.688)	0.654 (0.608-0.696)	0.695 (0.606-0.764)	0.647 (0.571-0.709)
DL-only (Late Fusion)	T1 + T1CE + T2	0.610 ± 0.020	0.604 (0.570-0.641)	0.623 (0.574-0.671)	0.558 (0.471-0.648)	0.574 (0.505-0.645)
Combined (Late Fusion)	T1 + T1CE + T2	0.680 ± 0.036	0.679 (0.644-0.710)	0.687 (0.644-0.730)	0.706 (0.626-0.777)	0.645 (0.564-0.710)
Clinical Only	T1 + T1CE + T2	0.654 ± 0.034	0.656 (0.624-0.688)	0.654 (0.608-0.696)	0.695 (0.606-0.764)	0.647 (0.571-0.709)
DL-only (CAF)	T1 + T1CE + T2	0.635 ± 0.019	0.629 (0.594-0.664)	0.624 (0.577-0.669)	0.620 (0.537-0.699)	0.649 (0.577-0.718)
Combined (CAF)	T1 + T1CE + T2	0.694 ± 0.023	0.691 (0.660-0.721)	0.688 (0.644-0.728)	0.716 (0.640-0.782)	0.689 (0.614-0.749)

CAF, cross-attention fusion; C-index, Harrell’s concordance index; CI, confidence interval; DL, deep learning; SD, standard deviation. Cross-validation C-index is presented as mean ± SD across 5 folds. Pooled and per-cohort C-indices are presented as point estimates with 95% CIs obtained from 1, 000 bootstrap resamples.

**Figure 4:**
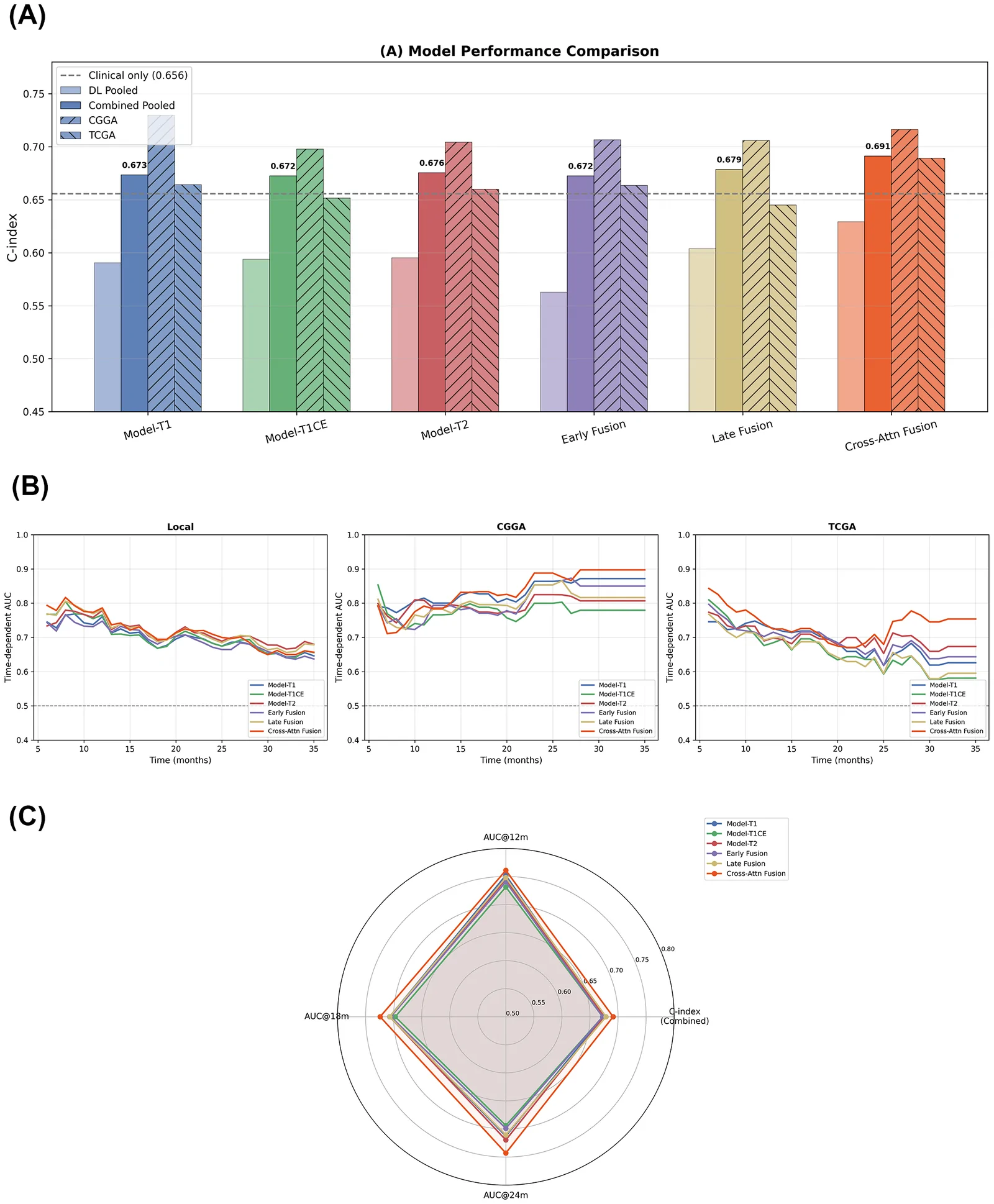
Discriminative performance comparison of the six deep learning models. **(A)** Grouped bar charts of Harrell’s C-index across four evaluation scopes (pooled, Local, CGGA, TCGA), with error bars representing the standard deviation of the five-fold cross-validation means. **(B)** Change curves of the time-dependent area under the receiver operating characteristic curve (td-AUC) over time (0–24 months), plotted by model. **(C)** A multidimensional performance radar chart comprehensively depicting the overall performance profile of each model based on five evaluation dimensions (pooled C-index, Local C-index, CGGA C-index, TCGA C-index, average td-AUC from 12–24 months). The td-AUC was calculated using the inverse probability of censoring weighting method. AUC, area under the curve; C-index, concordance index.

Among the three fusion architectures (**Table 2**; **Figure 4**), the CAF achieved the numerically highest average validation C-index (0.635 ± 0.019, pooled = 0.629), showing consistent performance trends above LF (0.610 ± 0.020, pooled = 0.604), EF (0.570 ± 0.032, pooled = 0.563), and all single-modality baselines. The C-indices for CAF across the three cohorts were 0.624 for Local, 0.620 for CGGA, and 0.649 for TCGA. The C-index for EF was lower than that of all three single-modality models. Because LF utilizes the same three-encoder backbone and concatenated representation as CAF but omits the cross-attention module (99.87 M vs. 106.18 M total parameters; **Supplementary Table 2**), it serves as a capacity-matched baseline. At this matched capacity, CAF achieved numerically higher discrimination than LF (DL-only C-index 0.635 ± 0.019 vs. 0.610 ± 0.020; pooled 0.629 vs. 0.604), suggesting that the numerical gain is associated with the cross-attention mechanism rather than model capacity alone. DeLong’s tests revealed that pairwise time-dependent AUC differences among models did not reach statistical significance (all *P* > 0.05; **Supplementary Figures 1**, **S2**), but the C-indices presented consistent trends across cohorts and time points.

### Incremental value of the combined model

The integration of deep learning risk scores with clinical variables further enhanced predictive performance (**Table 3**). Taking the CAF model as an example, the combined model (deep learning + clinical variables) yielded an average C-index of 0.694 ± 0.023 (pooled = 0.691, 95% CI: 0.660–0.721), which was superior to the clinical-variables-only model (0.654 ± 0.034, pooled = 0.656) and the deep-learning-only model (0.635 ± 0.019, pooled = 0.629). The combined model proved to be optimal in all cohorts: Local (combined 0.688 vs. clinical 0.654 vs. DL 0.624), CGGA (0.716 vs. 0.695 vs. 0.620), and TCGA (combined 0.689 vs. clinical 0.647 vs. DL 0.649). In the TCGA cohort, the deep-learning-only model slightly outperformed the clinical-variables-only model (0.649 vs. 0.647), whereas the clinical-variables-only model was superior to the deep-learning-only model in the other cohorts, suggesting variability in the relative contributions of imaging and clinical features to prognostic prediction across different cohorts. To match the information available at different clinical time points, we report this combined model in two configurations: a pre-treatment configuration restricted to variables available before treatment initiation (imaging score, age, sex, and MGMT status; pooled C-index 0.642), and a treatment-era full model additionally incorporating adjuvant therapy (pooled C-index 0.691; **Supplementary Table 9**). The full model serves as the primary model for the incremental-value and multivariable analyses below, whereas applicability before treatment planning is confined to the pre-treatment configuration.

**Table 3:** Time-dependent area under the receiver operating characteristic curve (AUC) for combined clinical + deep-learning models.

Model	Cohort	12-month AUC (95% CI)	18-month AUC (95% CI)	24-month AUC (95% CI)
Combined (T1)	Internal	0.761 (0.689-0.827)	0.668 (0.587-0.744)	0.683 (0.594-0.766)
Combined (T1)	CGGA	0.800 (0.681-0.898)	0.827 (0.716-0.922)	0.864 (0.769-0.944)
Combined (T1)	TCGA	0.735 (0.619-0.837)	0.704 (0.589-0.806)	0.663 (0.524-0.785)
Combined (T1)	Pooled	0.752 (0.699-0.802)	0.707 (0.652-0.762)	0.713 (0.646-0.775)
Combined (T1CE)	Internal	0.765 (0.696-0.829)	0.669 (0.591-0.746)	0.683 (0.593-0.771)
Combined (T1CE)	CGGA	0.766 (0.635-0.871)	0.788 (0.666-0.890)	0.800 (0.682-0.902)
Combined (T1CE)	TCGA	0.676 (0.544-0.773)	0.682 (0.566-0.779)	0.635 (0.504-0.749)
Combined (T1CE)	Pooled	0.731 (0.674-0.782)	0.697 (0.644-0.758)	0.694 (0.632-0.760)
Combined (T2)	Internal	0.779 (0.708-0.843)	0.690 (0.604-0.768)	0.700 (0.605-0.784)
Combined (T2)	CGGA	0.786 (0.659-0.894)	0.774 (0.641-0.892)	0.825 (0.722-0.920)
Combined (T2)	TCGA	0.690 (0.574-0.789)	0.696 (0.571-0.799)	0.698 (0.571-0.822)
Combined (T2)	Pooled	0.745 (0.690-0.796)	0.707 (0.649-0.764)	0.720 (0.659-0.779)
Combined (Early Fusion)	Internal	0.748 (0.674-0.813)	0.680 (0.601-0.755)	0.671 (0.578-0.760)
Combined (Early Fusion)	CGGA	0.794 (0.667-0.903)	0.771 (0.630-0.891)	0.853 (0.753-0.940)
Combined (Early Fusion)	TCGA	0.703 (0.585-0.806)	0.715 (0.606-0.813)	0.667 (0.542-0.790)
Combined (Early Fusion)	Pooled	0.741 (0.684-0.791)	0.705 (0.650-0.761)	0.699 (0.635-0.758)
Combined (Late Fusion)	Internal	0.781 (0.713-0.844)	0.687 (0.606-0.764)	0.695 (0.600-0.778)
Combined (Late Fusion)	CGGA	0.783 (0.660-0.885)	0.796 (0.665-0.898)	0.853 (0.761-0.934)
Combined (Late Fusion)	TCGA	0.692 (0.571-0.793)	0.686 (0.556-0.793)	0.642 (0.498-0.773)
Combined (Late Fusion)	Pooled	0.748 (0.693-0.799)	0.707 (0.653-0.767)	0.711 (0.646-0.776)
Combined (CAF)	Internal	0.786 (0.715-0.848)	0.694 (0.620-0.769)	0.709 (0.628-0.788)
Combined (CAF)	CGGA	0.784 (0.645-0.889)	0.834 (0.715-0.925)	0.888 (0.797-0.959)
Combined (CAF)	TCGA	0.739 (0.628-0.836)	0.709 (0.597-0.811)	0.709 (0.577-0.829)
Combined (CAF)	Pooled	0.761 (0.706-0.810)	0.724 (0.669-0.780)	0.743 (0.683-0.801)

AUC, area under the curve; CAF, cross-attention fusion; CI, confidence interval. Values are point estimates with 95% CIs obtained from 1, 000 bootstrap resamples.

**Figure 5:**
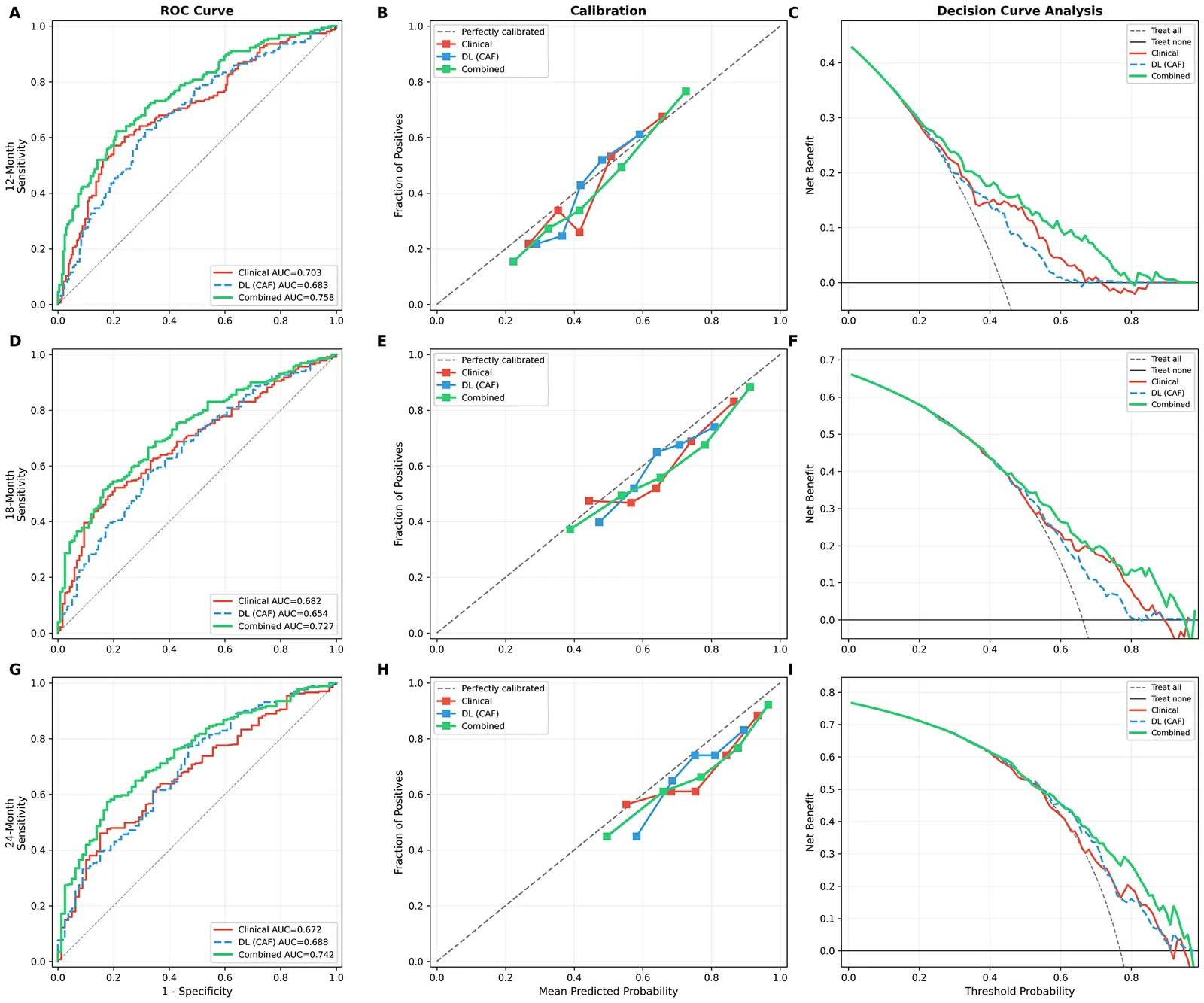
Comprehensive evaluation of discrimination, calibration, and clinical utility for the combined CAF model. This figure contains 9 subplots in a 3 rows × 3 columns layout. Rows correspond to the 12-, 18-, and 24-month time points, while columns correspond to discrimination (ROC), calibration, and decision curve analysis. **(A, D, G)** Time-dependent ROC curves (for 12, 18, and 24 months, respectively) comparing the discriminative performance of the clinical-variables-only, deep-learning-only (CAF), and combined models (deep learning + clinical variables) at the three time points, with the AUC values for each model annotated in the legend. **(B, E, H)** Calibration curves displaying the model predicted probability of events (X-axis) against the corresponding observed event fraction (Y-axis) for groups based on quantiles. The three models are connected by differently colored lines for grouped data points, and the black dashed line serves as the perfect calibration reference (y = x). **(C, F, I)** Decision curve analysis (DCA) presenting the net benefit curves for the combined model, clinical-variables-only model, and deep-learning-only model relative to “treat all” and “treat none” reference strategies. All predictions are based on out-of-fold predictions from five-fold cross-validation using the inverse probability of censoring weighting (IPCW) method. AUC, area under the curve; CAF, cross-attention fusion; DCA, decision curve analysis; IPCW, inverse probability of censoring weighting; ROC, receiver operating characteristic.

Time-dependent ROC analysis of the combined CAF model demonstrated consistent discriminative capability (**Figures 5A, D, G**), yielding pooled AUC values of 0.761 at 12 months, 0.724 at 18 months, and 0.743 at 24 months (average AUC for 12–24 months = 0.743). The institutional cohort achieved the highest 12-month AUC (0.786), whereas the CGGA cohort attained the highest AUCs at the later time points (0.834 at 18 months and 0.888 at 24 months; 0.784 at 12 months); the AUCs for the TCGA cohort ranged from 0.709 to 0.739 across time points. Calibration curves (**Figures 5B, E, H**) showed that the predicted probabilities of the combined model aligned well with the observed event rates at all three time points. Quantitatively (**Supplementary Table 11**), the combined CAF model demonstrated good calibration, with observed/expected ratios of 0.94 (95% CI: 0.83–1.04), 0.97 (95% CI: 0.90–1.05), and 0.98 (95% CI: 0.93–1.04), alongside calibration-in-the-large values of −0.07 (95% CI: −0.23–0.08), −0.07 (95% CI: −0.21–0.08), and −0.06 (95% CI: −0.21–0.10) at 12, 18, and 24 months, respectively. The corresponding calibration slopes were 1.29 (95% CI: 0.96–1.61), 0.92 (95% CI: 0.65–1.19), and 0.86 (95% CI: 0.56–1.16). At every evaluated time point, the O/E intervals included 1, the calibration-intercept intervals included 0, and the slope intervals included 1, indicating a lack of systematic miscalibration; calibration curves for all six deep learning models are provided in **Supplementary Figure 6**.

### Independent prognostic value and risk stratification

**Figure 6:**
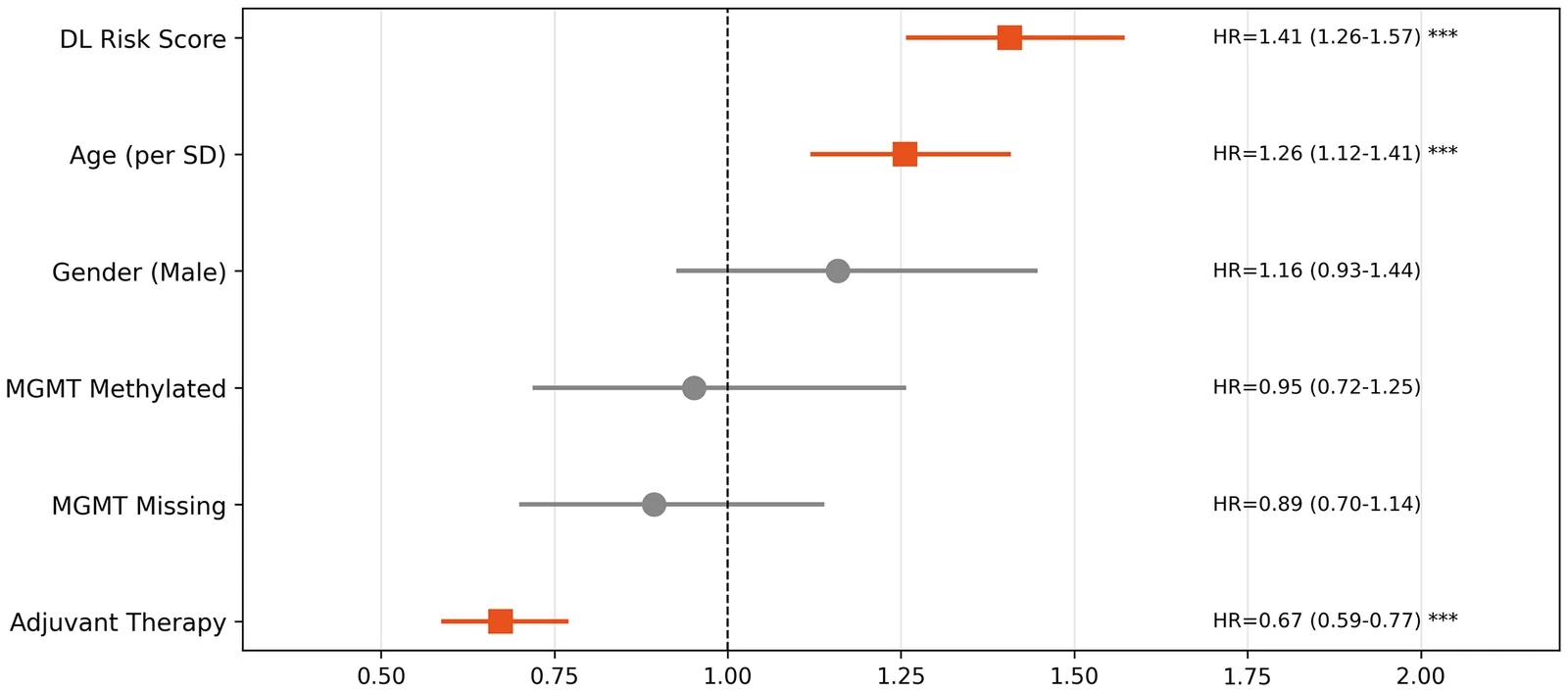
Forest plot of the multivariable Cox model (combined CAF model). Adjusted hazard ratios with 95% CIs are shown for the standardized deep learning risk score, age, gender, MGMT status (reference: unmethylated), and adjuvant therapy; the dashed line marks HR = 1 (no effect). Estimates are pooled across the five folds; corresponding values are listed in Table 4. CAF, cross-attention fusion; CI, confidence interval; HR, hazard ratio; MGMT, O⁶-methylguanine-DNA methyltransferase.

Multivariable Cox regression analysis (**Table 4**; **Figure 6**) demonstrated that, after adjusting for the available covariates (age, sex, MGMT status, and adjuvant therapy), the deep learning risk score was the strongest adverse prognostic factor (HR = 1.41, 95% CI: 1.26–1.57, *P* < 0.001), while adjuvant therapy was the strongest protective factor (HR = 0.67, 95% CI: 0.59–0.77, *P* < 0.001). Age also served as an independent prognostic factor (HR = 1.26, 95% CI: 1.12–1.41, *P* < 0.001). After adjusting for the deep learning risk score, gender (HR = 1.16, *P* = 0.19) and MGMT status (with unmethylated as the reference group: methylated HR = 0.95, *P* = 0.72; missing/Unknown HR = 0.89, 95% CI: 0.70–1.14) did not demonstrate independent prognostic value.

**Table 4:** Multivariable Cox proportional hazards regression analysis incorporating deep learning risk score and clinical covariates.

Variable	Hazard ratio (95% CI)	P-value
Age_std	1.26 (1.12-1.41)	<0.001
Gender	1.16 (0.93-1.44)	0.191
MGMT_Status	0.95 (0.72-1.25)	0.723
MGMT_missing	0.89 (0.70-1.14)	0.360
Adjuvant Therapy (Yes vs. No)	0.67 (0.59-0.77)	<0.001
dl_risk_std	1.41 (1.26-1.57)	<0.001

CI, confidence interval; HR, hazard ratio; MGMT, O^6^-methylguanine-DNA methyltransferase. The analysis includes a combined cohort of 386 patients using the Cross-Attention Fusion (CAF) risk score.

**Figure 7:**
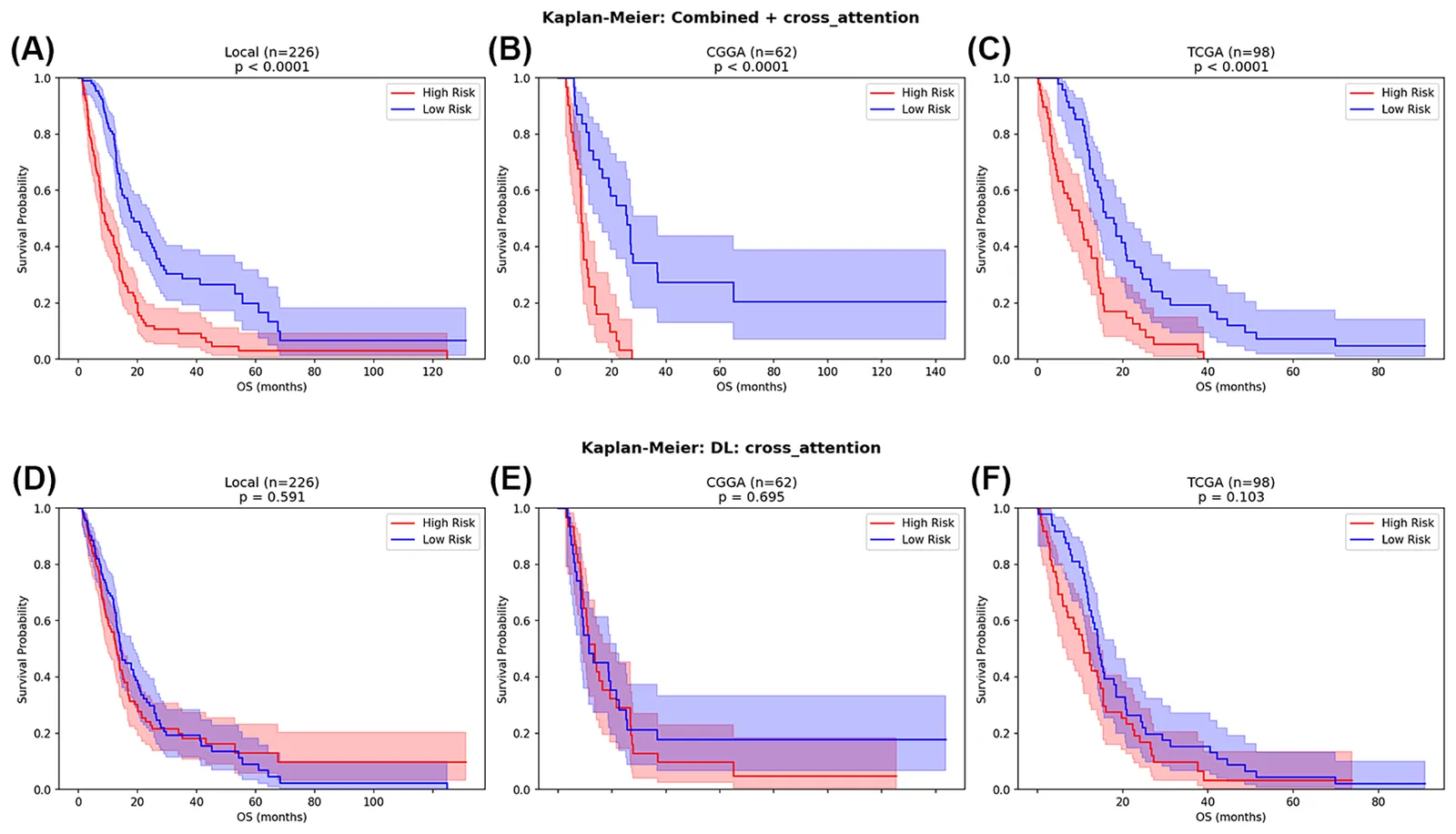
Kaplan-Meier survival analysis for the combined CAF model and the deep-learning-only CAF model. The figure follows a 2 rows × 3 columns layout. The top row **(A–C)** presents survival curves for the combined model (deep learning risk score + clinical variables integrated via Cox regression) in the Local cohort [**(A)**, n = 226], CGGA [**(B)**, n = 62], and TCGA [**(C)**, n = 98] cohorts, utilizing a binary stratification into high (red) and low (blue) risk groups based on the cohort-specific median of the out-of-fold (cross-validated) risk scores. All three cohorts exhibit significant intergroup separation (all log-rank *P* < 0.0001). The bottom row **(D–F)** depicts the survival curves for the deep-learning-only CAF model in the same three cohorts [**(D)** Local, **(E)** CGGA, **(F)** TCGA] using the identical stratification rule. None of the three cohorts achieved statistical significance (*P* = 0.591, 0.695, 0.103), underscoring the limitations of a single imaging score under fixed threshold division. Shaded areas represent 95% confidence intervals, and *P* values were derived from log-rank tests. CAF, cross-attention fusion.

Kaplan-Meier analysis further evaluated the risk stratification capability of the models (**Figure 7**; **Supplementary Figure 3**). The survival curves for the high-risk and low-risk groups defined by the combined CAF model exhibited significant separation in all three cohorts (all *P* < 0.0001; **Figures 7A–C**). Notably, although the deep learning risk score showed a significant independent prognostic effect as a continuous variable in multivariable Cox regression (HR = 1.41 per SD, *P* < 0.001), when it was converted to a binary classification using its median as a single cutoff, the deep-learning-only CAF model failed to achieve statistically significant risk stratification in any of the three cohorts (Local: *P* = 0.591; CGGA: *P* = 0.695; TCGA: *P* = 0.103; **Figures 7D–F**). This phenomenon highlights the limitations of utilizing a single imaging score under fixed threshold division, further supporting the necessity of integrating multidimensional clinical variables to achieve precise risk stratification (i.e., the combined CAF model). Among all six combined models, CAF achieved significant risk stratification in all cohorts, whereas the stratification effects of the other architectures were inconsistent (**Supplementary Figure 3**).

### Robustness and sensitivity analyses

The principal discrimination results were robust to our key methodological choices. Confining domain-adaptive pretraining to each training fold reproduced the global pipeline’s pooled out-of-fold C-index exactly (0.629 in both; *P* = 0.485; **Supplementary Table 4**), indicating that pretraining once on the full cohort introduced no detectable optimistic bias relative to a strictly fold-specific protocol. Discrimination was also essentially unchanged under realistic ±1-voxel mask perturbation—consistent with the high segmentation quality against expert ground truth (whole-tumor Dice 0.91–0.94; **Supplementary Tables 1**, **S5**)—confirming insensitivity to plausible segmentation error. Adding a FLAIR encoder did not improve the three-sequence model (ΔC-index = −0.021, *P* = 0.13; **Supplementary Table 6**), and the multivariable hazard ratios were stable under cohort-stratified re-estimation, consistent with the proportional-hazards assumption (deep learning risk score HR = 1.42 per SD; **Supplementary Table 7**).

This independence proved equally durable under more demanding covariate and missing-data conditions. In the institutional cohort, the score remained independently prognostic after further adjustment for the two prognostic factors unavailable in the public cohorts—extent of resection and KPS; although attenuated relative to the primary model (HR 1.19 vs 1.41 per SD, *P* = 0.021; **Supplementary Table 8**), it retained significance, indicating that its value is not merely a proxy for these unmeasured surgical and functional factors. In a strictly pre-treatment model excluding adjuvant therapy, the score likewise remained prognostic (HR 1.39 per SD, *P* < 0.001), with discrimination expectedly lower than the treatment-era full model (pooled C-index 0.642 vs 0.691; **Supplementary Table 9**), supporting its applicability at the time of diagnosis. Finally, in a complete-case analysis restricted to the 206 patients with documented MGMT status, MGMT itself remained non-significant (HR 0.99, *P* = 0.92)—comparable to the imputed full-cohort estimate—while the deep learning score stayed independently prognostic (HR 1.18, *P* = 0.030; **Supplementary Table 10**), suggesting that the null MGMT effect is unlikely to be driven primarily by the missing-data handling.

To provide an approximate external validation in which each cohort was entirely excluded from the survival-modeling pipeline, we performed a LOCO analysis (**Supplementary Table 14**). Under this design, the combined CAF model achieved C-indices of 0.671 (held-out Local), 0.708 (held-out CGGA), and 0.632 (held-out TCGA). Although discrimination was attenuated relative to the pooled cross-validation—consistent with the expected optimism of merged-cohort training—Kaplan–Meier risk stratification using the training-defined median cutoff remained statistically significant in all three held-out cohorts (log-rank *P* < 0.001, < 0.001, and 0.007, respectively). The deep-learning-only model was the component most sensitive to domain shift (C-index 0.56–0.59), indicating that the imaging feature extractor alone does not fully transfer across cohorts and that the clinical anchor contributes substantially to cross-cohort stability.

**Figure 8:**
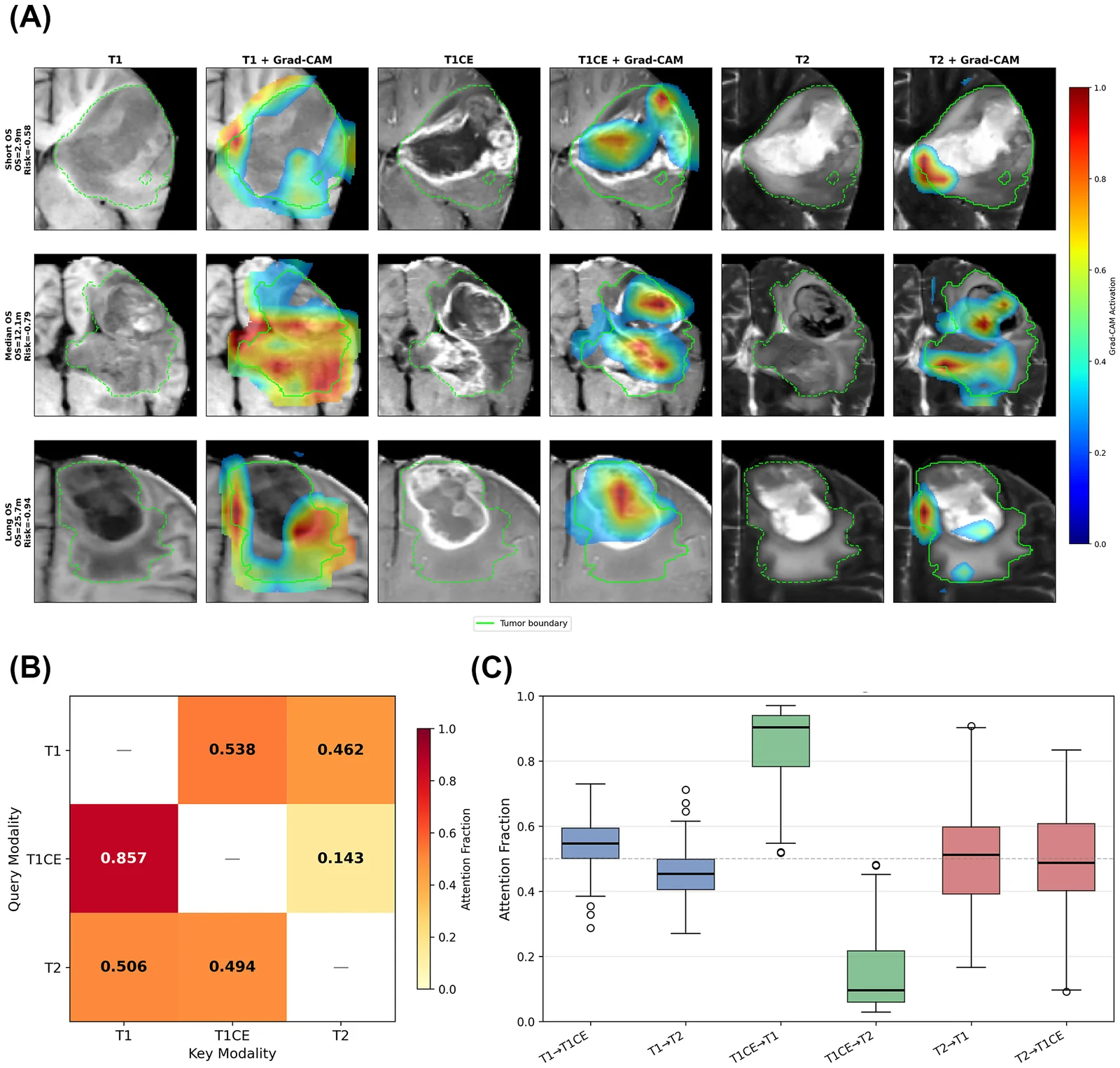
Interpretability analysis of the combined CAF model. **(A)** Grad-CAM visualization overlaying the Grad-CAM activation maps onto the original T1, T1CE, and T2 three-modality images for three representative patients corresponding to short, median, and long survival times. Activation intensity is encoded using a jet colormap (blue = low, red = high), and tumor boundaries (automatically segmented by nnU-Net) are delineated by green dashed lines. **(B)** Heatmap of the mean cross-modal attention weight matrix, where rows represent Query modalities and columns represent Key modalities. Cell values indicate the average attention scores for each modality pair (row-normalized, with non-applicable values on the diagonal). Darker colors denote higher attention weights. **(C)** Boxplot illustrating the patient-level distribution of cross-modal attention scores across the six ordered modality pairs (T1→T1CE, T1→T2, T1CE→T1, T1CE→T2, T2→T1, T2→T1CE) for all 386 patients. Boxes depict the interquartile range, center lines denote the median, whiskers extend to 1.5 × IQR, and outliers are individually marked. The gray dashed line (0.5) serves as a reference, revealing inter-patient heterogeneity in attention allocation. CAF, cross-attention fusion; Grad-CAM, Gradient-weighted Class Activation Mapping; IQR, interquartile range.

A range of exploratory analyses was concordant with the primary findings. The combined model’s potential clinical utility was supported by a greater net benefit on decision-curve analysis (**Figures 5C, F, I**) and by favorable Youden-based diagnostic metrics and Integrated Brier Scores (**Supplementary Tables 12**, **S13**), and its discrimination remained consistent across age, sex, and MGMT subgroups (**Supplementary Figure 5**). In a locally fitted configuration—refitting only the Cox layer on the institutional cohort and inferring on CGGA and TCGA—discrimination was attenuated, yet binary risk stratification with a fixed institutional cutoff remained significant in both external cohorts; because both contributed to feature-extractor training, this analysis is hypothesis-generating rather than a formal external validation (**Supplementary Figure 7**; **Supplementary Table 16**). Analyses of the model itself were equally supportive: dual-modality ablation confirmed the benefit of using all three sequences (**Supplementary Table 15**), Uniform Manifold Approximation and Projection (UMAP) (28) projections showed no dominant center-driven clustering, although this qualitative projection cannot formally exclude batch effects (**Supplementary Figure 4**), and Grad-CAM (29) and cross-attention maps showed spatial alignment with tumor regions, offering preliminary insight into the model’s focus (**Figure 8**).

## Discussion

Under a multicenter pooled training, unified backbone, and two-stage paradigm, this study conducted a systematic head-to-head comparison of three multimodal MRI deep learning survival prediction architectures—EF, LF, and CAF—and quantified their incremental value when integrated with routine clinical variables. The three main contributions of this study can be summarized as follows. First, the application of a unified 3D ResNet-18 backbone controlled for the backbone heterogeneity confounding commonly seen in prior multimodal studies. Second, a multicenter design comprising 386 patients from our institution, CGGA, and TCGA enabled the evaluation of cross-cohort transferability of the fusion strategies under both pooled cross-validation and a LOCO external validation. Third, a comprehensive performance evaluation encompassing discrimination and calibration was performed using the C-index, time-dependent AUC, and Integrated Brier Score, complemented by an exploratory assessment of potential clinical utility (Youden-based diagnostic metrics and decision curve analysis). On the primary C-index metric, CAF showed numerically higher and consistent performance than early and LF, and time-dependent AUCs trended favorably across most cohorts and time points. However, pairwise AUC differences were not statistically significant (DeLong, all *P* > 0.05); this pattern therefore represents a consistent numerical trend rather than statistically confirmed superiority. Upon integration with clinical variables, the combined CAF model showed improved and consistent discrimination across the three constituent cohorts under pooled cross-validation, and retained significant risk stratification in all held-out cohorts under LOCO validation. After adjusting for other covariates, the deep learning risk score emerged as the strongest adverse prognostic factor, alongside adjuvant therapy as the most potent protective factor.

Situating these findings within the specific context of previous literature further highlights their methodological and performance positioning. Within traditional radiomics, Bae et al. (30) integrated multiparametric MRI radiomics with clinical and gene expression profiles for GBM survival prediction, confirming that radiomic features can provide independent prognostic information. The deep learning results of our study are consistent with this observation, as the C-indices for the single-modality T1, T1CE, and T2 models performed similarly, indicating no significant advantage for any single modality. Nevertheless, this study further indicates that the method of feature integration, specifically the selection of the fusion architecture, is more important than the choice of individual modalities. Without increasing the number of input modalities, CAF elevated the C-index from approximately 0.59 for single modalities to 0.629 (DL-only), and reached 0.691 after clinical variable integration.

In the domain of deep learning for multimodal MRI survival prediction, Nie et al. (31) were early adopters of 3D deep learning for predicting survival times in high-grade gliomas from multimodal MRI, laying the methodological groundwork for this direction. Yoon et al. (32) developed a DL survival prediction model based on multiparametric MRI for GBM patients who had undergone surgery and concurrent chemoradiotherapy. The DeepRisk model proposed by Li et al. (33) predicted glioma survival directly from whole-brain MRI without requiring tumor segmentation, demonstrating independent incremental prognostic value relative to traditional clinical variables. A Transformer-based multimodal deep learning survival prediction model reported by Gomaa et al. (11) integrated complementary information from various input modalities and displayed robust cross-institutional generalization performance. Luckett et al. (34) further confirmed that relying solely on preoperative multimodal neuroimaging features can achieve GBM survival stratification independently of surgical, treatment, and molecular information, underscoring the value of imaging biological information as an independent prognostic dimension. Recently, Akbari et al. (35) validated the robustness of GBM prognostic subtyping based on imaging and clinical features in a large-scale cohort using a multicenter machine learning architecture. Compared to these prior studies, the uniqueness of our research lies in the systematic comparison of three fusion architectures under a unified backbone and the demonstration of consistent cross-cohort performance under both pooled cross-validation and LOCO external validation. Methodologically, these consistent trends support CAF as a promising candidate architecture for multimodal MRI survival prediction, although confirmation in larger cohorts with adequate statistical power will be required to establish formal superiority. A noteworthy derivative finding is that EF yielded the poorest performance in this study (C-index = 0.563), underperforming both LF (0.604) and CAF (0.629), as well as all three single-modality baselines (0.590–0.595). This result suggests caution for studies favoring Transformer-like architectures that rely on input-layer channel concatenation, as simplistic EF might dilute modality-specific representations and consequently impair predictive performance. Thus, this finding advocates for design paradigms that utilize independent modality encoding alongside explicit cross-modal attention interactions.

Because CAF and LF share an identical, capacity-matched backbone (differing only by the cross-attention module), the numerical advantage of CAF most plausibly reflects explicit modeling of inter-modality dependencies rather than merely a larger parameter budget. By contrast, the lower performance of EF should be interpreted cautiously, as its single-encoder design and heuristic stem initialization confound the fusion strategy with constrained model capacity.

The performance advantages of CAF may tentatively be interpreted in light of the multi-regional biological heterogeneity of GBM. The three MRI modalities each carry complementary tumor biological information. T1CE reflects contrast leakage, fundamentally serving as an imaging manifestation of blood-brain barrier (BBB) disruption and tumor neoangiogenesis. GBM induces abnormal neoangiogenesis through the overexpression of angiogenic factors such as vascular endothelial growth factor (VEGF), and defects in tight junctions and incomplete basement membranes in these vessels allow contrast agents to leak into the tumor interstitium, forming enhanced regions (36). T2/FLAIR hyperintensity, by contrast, reflects peritumoral edema, yet this region is biologically heterogeneous, containing both vasogenic edema and active tumor cell infiltration. Rathore et al. (37) and Prasanna et al. (38) respectively confirmed that radiomic features of peritumoral edema regions can predict recurrence patterns and significantly differentiate between long- and short-term surviving GBM patients, indicating that T2 abnormal regions carry important prognostic signals. Unenhanced T1 provides a baseline anatomical reference. “Imaging habitat” analyses based on multiparametric MRI have further revealed the presence of subregions with distinct biological behaviors within GBM, with spatial distributions significantly correlated with patient prognosis (39, 40). Recent studies have also corroborated that combining habitat analysis with deep learning features can optimize glioma grading and molecular status prediction performance (41, 42), thereby supporting tumor subregion heterogeneity as a source of prognostic information. These investigations provide the biological rationale for the CAF architecture utilized in this study: different modalities capture distinct biological dimensions of the tumor, and cross-modal interactions can explicitly model the dependencies among these dimensions.

The correspondence between attention weight patterns and biological phenotypes (**Figures 8B, C**) is qualitatively consistent with this hypothesis, although it does not by itself establish the underlying mechanism. The highest average attention on T1CE aligns with the enhancing tumor core as a central prognostic signal, whereas the substantial between-patient heterogeneity echoes spatial phenotypic diversity of GBM described in the aforementioned imaging habitat research, suggesting that the model does not mechanically integrate modalities with fixed weights, but rather dynamically adjusts modality emphasis based on the individual imaging biological characteristics of each patient. A concrete example illustrates this mechanism. In GBM subtypes characterized primarily by diffuse non-enhancing infiltration, the enhancing information on T1CE is relatively limited. In such cases, CAF may automatically reduce its reliance on T1CE, shifting higher attention weights to regions of T2 abnormal hyperintensity to capture occult tumor burden and invasive margins. This pattern is qualitatively consistent with biological observations by Sarkaria et al. (43) that BBB disruption is not present in all GBM regions and that the BBB may remain relatively intact in some infiltrative tumor areas. These attention-based interpretations are exploratory and hypothesis-generating, and should not be construed as causal or histopathologically validated evidence. The cross-modal query mechanism of CAF may allow the model to draw on the infiltrative features of T2 in regions with limited T1CE enhancement, complementing a single enhancing modality.

The potential clinical value of this study lies primarily in providing a noninvasive, accessible, and independent information dimension based on routine MRI for individualized GBM prognostic evaluation. Multivariable Cox regression showed that the deep learning risk score retained independent prognostic value after adjusting for the available covariates (HR = 1.41, *P* < 0.001), representing, alongside adjuvant therapy, one of the two most significant independent prognostic factors in this study, thereby supporting the meaningful supplementary role of imaging-derived information to traditional clinical scales. A local-cohort sensitivity analysis further confirmed that this independence persisted after additionally adjusting for extent of resection and KPS. This value is particularly prominent in specific clinical scenarios. First, when primary care hospitals or regional medical centers struggle to obtain immediate molecular subtyping information due to a lack of standardized MGMT methylation testing capabilities, the imaging-derived risk score—derived solely from preoperative MRI—can serve as a practically feasible adjunct for preoperative individualized evaluation; this preoperative applicability pertains to the imaging-derived score itself and to the pre-treatment combined model, whereas the full combined model incorporating adjuvant therapy is appropriately positioned as a treatment-era prognostic tool. Second, in emergency or urgent assessment scenarios, there is often a 2–3 week window from the initial MRI to the availability of the molecular pathology report. During this period, before treatment decisions are finalized, the imaging-derived score can provide a prognosis-oriented auxiliary basis within this critical window. Third, in the context of rapid stratification for multicenter clinical trials, candidate patients can be preliminarily screened based on routine MRI for prognosis-oriented trial entry before pathological results are returned, thereby enhancing the efficiency of trial enrollment.

Compared to established prognostic tools primarily based on clinical variables, such as the RTOG-RPA (44) and the Gittleman Nomogram (6), the complementary value of our model in specific clinical contexts is primarily demonstrated in two aspects. First, in realistic clinical scenarios characterized by incomplete information on molecular markers such as MGMT (with a 46.6% missing rate for MGMT status in this study), an imaging-derived risk score based on routine preoperative MRI can function as a supplementary prognostic dimension that is available at the time of diagnosis, independently of any subsequent treatment information. The prognostic value of MGMT methylation status as a crucial molecular marker for GBM was established by the landmark study of Hegi et al. (8). Nevertheless, its clinical accessibility is constrained by factors such as testing facility availability and specimen quality, a gap that an imaging-derived score can partially bridge. The observation that MGMT lacked independent prognostic value in the primary multivariable model (HR = 0.95, *P* = 0.72) must be interpreted with caution given this missing rate. A complete-case sensitivity analysis restricted to the 206 patients with documented MGMT status showed that MGMT remained non-significant when entered directly without imputation (HR = 0.99, *P* = 0.92; **Supplementary Table 10**), suggesting that the null finding is unlikely to be primarily an artifact of the missing-indicator strategy and may instead reflect this cohort’s treatment composition and the limited statistical power for a single binary marker after multivariable adjustment. Importantly, this null result in no way contradicts the well-established prognostic and predictive significance of MGMT methylation; accordingly, the imaging-derived risk score is intended strictly as a complementary prognostic dimension—not a replacement or surrogate for MGMT testing—that may provide added information particularly when standardized molecular testing is unavailable. Notably, the deep learning risk score retained independent prognostic significance in the complete-case model (HR = 1.18, *P* = 0.030), confirming that its prognostic contribution does not depend on the MGMT missing-data handling approach. Second, the model achieved high positive predictive values (0.87–0.92) under the Youden cutoffs at 18 and 24 months (**Supplementary Table 12**), suggesting its particular utility in identifying high-risk individuals. For instance, it could serve as a reference in multidisciplinary discussions for identifying candidates necessitating more intensive follow-up or clinical trial enrollment. The observation of relatively low sensitivity (0.50–0.60) implies that this cutoff is not suited for broad-spectrum screening but should rather be focused on high-risk decision-making scenarios demanding high specificity. We did not re-derive the RTOG-RPA or Gittleman nomogram on this dataset; accordingly, we do not claim numerical superiority over these established tools. Instead, it emphasizes the complementary value of the deep learning risk score as an orthogonal imaging biological dimension to existing clinical tools. This positions the model as complementary rather than alternative to traditional tools and provides a basis for developing integrated prognostic frameworks.

A noteworthy observation is that the combined CAF model exhibited a higher C-index (0.716, 95% CI: 0.640–0.782) and 24-month AUC (0.888, 95% CI: 0.797–0.959) on the CGGA cohort compared to our institutional cohort (C-index 0.688, 95% CI: 0.644–0.728; 24-month AUC 0.709, 95% CI: 0.628–0.788). This contradicts the typical pattern where performance is superior on the primary training cohort. Because this study employed a multicenter pooled training design with stratified cross-validation within folds, both the CGGA and TCGA cohorts participated in model training. Consequently, the aforementioned comparison is not an internal versus external validation in the traditional sense, but rather a performance comparison among different cohorts within a multicenter pooled framework. Potential sources for this discrepancy include the event rate in the CGGA cohort (87.1%), which was significantly higher than that of the Local cohort (74.8%); a higher event density favors the statistical stability of the C-index and facilitates the capture of survival differences by the risk score. Moreover, the adjuvant therapy rate in the CGGA cohort (93.5%) was considerably higher than that of the Local cohort (69.0%), and a more homogeneous distribution of treatment status as a covariate mitigates interference from unobserved confounders. Furthermore, the institutional cohort comprised 58.5% of the total sample, which may have introduced greater patient heterogeneity across different acquisition periods and scanner types, whereas CGGA, being a publicly curated dataset, possesses relatively consistent scanning parameters. These cross-cohort differences in C-index more likely reflect inherent disparities in predictability driven by event rates and treatment homogeneity, rather than a model bias toward any specific cohort. Consistent with this, CAF’s advantage in C-index was stable across cohorts and time points rather than confined to any single one, supporting it as a stable—if not yet statistically confirmed—integration strategy.

### Limitations

There are several limitations to this study. First, regarding data structure, the retrospective design may introduce selection bias, and the institutional cohort accounts for 58.5% (226/386) of the pooled sample, which may bias the model toward the Local cohort’s data distribution. The total of 386 cases, although typical for GBM research, remains modest for deep learning. Relatedly, the combined model’s discrimination was moderate in absolute terms (pooled C-index 0.69), and the pairwise differences among the fusion architectures in time-dependent AUC did not reach statistical significance; the preference for CAF therefore rests on consistent C-index trends rather than on definitive superiority.

Second, regarding clinical covariates, the multivariable Cox model incorporated only four routine variables (age, gender, MGMT methylation status, and adjuvant therapy). EOR and KPS were not systematically recorded in the public cohorts; in a local-cohort sensitivity analysis additionally adjusting for both, the deep learning risk score remained independently prognostic, although confirmation in multicenter cohorts with complete EOR and KPS data is still required. MGMT methylation status was missing for 46.6% of patients and was handled with a missing-indicator approach; a complete-case sensitivity analysis indicated that the null MGMT finding was unlikely to be solely an artifact of this strategy; nonetheless, the high rate of missingness and the limited number of documented cases markedly constrain the power to characterize the true MGMT effect, so this null result should be interpreted with caution, without diminishing the established clinical importance of MGMT methylation. Because adjuvant therapy becomes available only after treatment planning, the full combined model is a treatment-era rather than a strictly preoperative tool; we therefore also fitted a pre-treatment configuration (age, sex, MGMT status, and the imaging-derived risk score), to which our claims of pre-treatment applicability are confined.

Third, regarding sequence completeness, FLAIR was unavailable in CGGA, so the model used only the three routine sequences (T1, T1CE, T2). FLAIR is clinically important for delineating non-enhancing tumor burden, infiltrative margins, and peritumoral edema, and its omission may limit the features the model can capture; we do not claim that T2 or the cross-attention mechanism biologically compensates for this loss. In an exploratory sub-analysis within the FLAIR-available subcohort, appending a FLAIR encoder did not improve discrimination, but this is not biological evidence against FLAIR, as the FLAIR stream lacked a segmentation-pretrained encoder and was tested in a smaller subcohort. Properly exploiting FLAIR—with a dedicated pretrained encoder and complete multicenter data—remains important future work; recent robust pipelines for missing modalities (45) offer a pathway for deployment across centers with incomplete sequences.

Fourth, regarding generalization, because all three cohorts contributed to training within each fold, the per-cohort results reflect internal multicenter cross-validation rather than external validation. A LOCO analysis, in which each cohort was entirely withheld, showed attenuated discrimination but significant risk stratification in all held-out cohorts; nonetheless, LOCO is not prospective validation on an independent, temporally and geographically distinct population, and the modest cohort sizes (particularly CGGA, n = 62) limit precision. Strict prospective external validation therefore remains necessary before clinical deployment.

Fifth, regarding interpretability, the Grad-CAM maps and cross-attention weights are exploratory, *post-hoc*, association-based explanations; they indicate where the model attends but do not establish biological causality or histopathological correspondence. Whether individual attention heads stably map to specific cross-modal associations, and whether such patterns are reproducible across tumor subtypes, remains to be established through dedicated attention-probing methods, spatially co-localized multi-regional histopathology, or mechanistic interpretability tools—a shared challenge for attention-based fusion methods and a focus for future work.

Finally, regarding preprocessing and clinical translation, the pipeline relied on N4 correction, ANTs registration, skull stripping, and nnU-Net segmentation. The automated segmentations were visually inspected by a neuroradiologist without gross failures, and a mask-perturbation analysis confirmed robustness to realistic boundary variations; however, nnU-Net was not fine-tuned on this multicenter cohort, so routine visual quality control is recommended for future deployments. In addition, Stage-1 domain-adaptive pretraining was performed globally on all 386 patients before cross-validation; although no survival labels were used, validation-fold images contributed to representation learning, which could in principle introduce optimistic bias in a small dataset—a fold-specific sensitivity analysis showed this effect to be negligible, but fully nested pretraining should be standard in future prospective work. More broadly, genuine clinical deployment will require prospective independent validation, real-world robustness evaluation, regulatory clearance, workflow integration, and acceptance of models with limited interpretability.

To surmount these limitations and drive clinical translation, future research should focus on the following directions. Prospective multicenter validations employing standardized imaging protocols should be conducted. Diffusion-weighted, perfusion, and spectroscopic imaging should be integrated to enrich the dimensions of tumor biology. Foundation models should be explored to leverage large-scale pretraining for improved feature representations. Recent studies have already preliminarily confirmed the feasibility of medical imaging foundation models in assisting brain tumor diagnosis and molecular status prediction, and a generalizable foundation model for brain MRI published in 2026 further demonstrated cross-task transfer capabilities (46, 47). Multi-task learning frameworks should be developed to jointly predict overall survival, progression-free survival, and molecular markers such as MGMT methylation and telomerase reverse transcriptase (TERT) promoter mutations. Clinical utility studies must be performed to evaluate the actual impact of model predictions on decisions such as treatment intensity selection and follow-up density adjustments in simulated decision-making contexts, rather than relying solely on statistical metrics. Finally, methods for integrating deep learning risk scores with traditional tools like RTOG-RPA should be explored. Through these efforts, multimodal deep learning may develop from current methodological foundations into integrated prognostic frameworks that combine imaging, clinical, and molecular information, ultimately supporting more precise and individualized clinical decisions for GBM, which remains one of the most prognostically challenging tumors in neuro-oncology.

## Conclusion

Under a unified deep learning framework, this multicenter study systematically compared three multimodal MRI fusion architectures for IDH-wildtype GBM survival prediction. Across cohorts, CAF yielded numerically higher and consistent performance than early and late fusion under both pooled cross-validation and LOCO external validation, although pairwise differences in time-dependent AUC were not statistically significant. By explicitly modeling the nonlinear dependencies among modalities and dynamically allocating modality weights at the individual patient level, this architecture aligns with the imaging heterogeneity of GBM. Following integration with routine clinical variables, the imaging-derived risk score—derived solely from preoperative MRI—retained independent prognostic value after adjustment for the available clinical and molecular covariates. The score may therefore serve as a complementary prognostic tool alongside traditional clinical factors, particularly where molecular information such as MGMT is limited. Rather than establishing the superiority of any single architecture, this study provides a fair, backbone-controlled basis for comparing multimodal MRI fusion strategies and identifies CAF as a promising candidate; prospective multicenter external validation is required before clinical translation in individualized risk stratification for GBM.

## Data Availability

The original contributions presented in the study are included in the article/**Supplementary Material**. Further inquiries can be directed to the corresponding author.

## References

[1] Louis DN Perry A Wesseling P Brat DJ Cree IA Figarella-Branger D . The 2021 WHO classification of tumors of the central nervous system: a summary. Neuro-Oncol. (2021) 23:1231–51. doi: 10.1093/neuonc/noab106 34185076 PMC8328013

[2] Stupp R Mason WP Van Den Bent MJ Weller M Fisher B Taphoorn MJB . Radiotherapy plus concomitant and adjuvant temozolomide for glioblastoma. N Engl J Med. (2005) 352:987–96. doi: 10.1056/nejmoa043330 15758009

[3] Fukushima CM de Groot J . Updates for newly diagnosed and recurrent glioblastoma: a review of recent clinical trials. Curr Opin Neurol. (2024) 37:666–71. doi: 10.1097/WCO.0000000000001320 39258745 PMC11540275

[4] Aboubakr O Moiraghi A Elia A Tauziede-Espariat A Roux A Leclerc A . Long-term survivors in 976 supratentorial glioblastoma, IDH-wildtype patients. J Neurosurg. (2025) 142:174–86. doi: 10.3171/2024.5.JNS24393 39213667

[5] Ostrom QT Price M Neff C Cioffi G Waite KA Kruchko C . CBTRUS statistical report: primary brain and other central nervous system tumors diagnosed in the United States in 2016-2020. Neuro-Oncol. (2023) 25:iv1–iv99. doi: 10.1093/neuonc/noad149 37793125 PMC10550277

[6] Gittleman H Lim D Kattan MW Chakravarti A Gilbert MR Lassman AB . An independently validated nomogram for individualized estimation of survival among patients with newly diagnosed glioblastoma: NRG oncology RTOG 0525 and 0825. Neuro-Oncol. (2017) 19:669–77. doi: 10.1093/neuonc/now208 28453749 PMC5464437

[7] Eid M Alset D Timoshkina N Gvaldin D Rostorguev E Kavitskiy S . IDH mutation and MGMT methylation status in glioblastoma and other gliomas patients: a Russian retrospective cohort study. J Egypt Natl Cancer Inst. (2025) 37:36. doi: 10.1186/s43046-025-00296-w 40377745 PMC13313434

[8] Hegi ME Diserens A-C Gorlia T Hamou M-F De Tribolet N Weller M . MGMT gene silencing and benefit from temozolomide in glioblastoma. N Engl J Med. (2005) 352:997–1003. doi: 10.1056/NEJMoa043331 15758010

[9] Yang Z Zamarud A Marianayagam NJ Park DJ Yener U Soltys SG . Deep learning-based overall survival prediction in patients with glioblastoma: an automatic end-to-end workflow using pre-resection basic structural multiparametric MRIs. Comput Biol Med. (2025) 185:109436. doi: 10.1016/j.compbiomed.2024.109436 39637462 PMC11761382

[10] Kim G Xing F Kong H-J Santarnecchi E Shih HA Bortfeld T . Overall survival prediction of brain tumor patients with multimodal mri using swin unetr. Proc IEEE Int Symp BioMed Imaging. (2025) 2025, 1–5. doi: 10.1109/isbi60581.2025.10981128 40809516 PMC12345447

[11] Gomaa A Huang Y Hagag A Schmitter C Höfler D Weissmann T . Comprehensive multimodal deep learning survival prediction enabled by a transformer architecture: a multicenter study in glioblastoma. Neuro-Oncol Adv. (2024) 6:vdae122. doi: 10.1093/noajnl/vdae122 39156618 PMC11327617

[12] Li Y Xu W Zhao C Zhang J Zhang Z Shen P . Survival risk stratification of 2021 WHO glioblastoma by MRI radiomics and biological exploration. BMC Cancer. (2025) 25:1505. doi: 10.1186/s12885-025-14906-2 41044660 PMC12495617

[13] Fathi Kazerooni A Saxena S Toorens E Tu D Bashyam V Akbari H . Clinical measures, radiomics, and genomics offer synergistic value in AI-based prediction of overall survival in patients with glioblastoma. Sci Rep. (2022) 12:8784. doi: 10.1038/s41598-022-12699-z 35610333 PMC9130299

[14] Huang S-C Pareek A Seyyedi S Banerjee I Lungren MP . Fusion of medical imaging and electronic health records using deep learning: a systematic review and implementation guidelines. NPJ Dig Med. (2020) 3:136. doi: 10.1038/s41746-020-00341-z 33083571 PMC7567861

[15] Patel AP Tirosh I Trombetta JJ Shalek AK Gillespie SM Wakimoto H . Single-cell RNA-seq highlights intratumoral heterogeneity in primary glioblastoma. Science. (2014) 344:1396–401. doi: 10.1126/science.1254257 24925914 PMC4123637

[16] Verhaak RGW Hoadley KA Purdom E Wang V Qi Y Wilkerson MD . Integrated genomic analysis identifies clinically relevant subtypes of glioblastoma characterized by abnormalities in PDGFRA, IDH1, EGFR, and NF1. Cancer Cell. (2010) 17:98–110. doi: 10.1016/j.ccr.2009.12.020 20129251 PMC2818769

[17] Zhao Z Zhang K-N Wang Q Li G Zeng F Zhang Y . Chinese glioma genome atlas (CGGA): a comprehensive resource with functional genomic data from chinese glioma patients. Genomics Proteomics Bioinf. (2021) 19:1–12. doi: 10.1016/j.gpb.2020.10.005 33662628 PMC8498921

[18] Baid U Ghodasara S Mohan S Bilello M Calabrese E Colak E . The RSNA-ASNR-MICCAI BraTS 2021 benchmark on brain tumor segmentation and radiogenomic classification. (2021). doi: 10.48550/arXiv.2107.02314

[19] Tustison NJ Avants BB Cook PA Zheng Y Egan A Yushkevich PA . N4ITK: improved N3 bias correction. IEEE Trans Med Imag. (2010) 29:1310–20. doi: 10.1109/TMI.2010.2046908 20378467 PMC3071855

[20] Avants BB Epstein CL Grossman M Gee JC . Symmetric diffeomorphic image registration with cross-correlation: evaluating automated labeling of elderly and neurodegenerative brain. Med Img Anal. (2008) 12:26–41. doi: 10.1016/j.media.2007.06.004 17659998 PMC2276735

[21] Isensee F Jaeger PF Kohl SAA Petersen J Maier-Hein KH . nnU-net: a self-configuring method for deep learning-based biomedical image segmentation. Nat Methods. (2021) 18:203–11. doi: 10.1038/s41592-020-01008-z 33288961

[22] Bakas S Akbari H Sotiras A Bilello M Rozycki M Kirby JS . Advancing the cancer genome atlas glioma MRI collections with expert segmentation labels and radiomic features. Sci Data. (2017) 4:170117. doi: 10.1038/sdata.2017.117 28872634 PMC5685212

[23] Chen S Ma K Zheng Y . Med3D: transfer learning for 3D medical image analysis. (2019). doi: 10.48550/arXiv.1904.00625

[24] Groenwold RHH White IR Donders ART Carpenter JR Altman DG Moons KGM . Missing covariate data in clinical research: when and when not to use the missing-indicator method for analysis. CMAJ: Can Med Assoc J = J Assoc Med Can. (2012) 184:1265–9. doi: 10.1503/cmaj.110977 22371511 PMC3414599

[25] Harrell FE Lee KL Mark DB . Multivariable prognostic models: issues in developing models, evaluating assumptions and adequacy, and measuring and reducing errors. Stat Med. (1996) 15:361–87. doi: 10.1002/(SICI)1097-0258(19960229)15:43C361::AID-SIM1683E3.0.CO;2-4 8668867

[26] Vickers AJ Elkin EB . Decision curve analysis: a novel method for evaluating prediction models. Med Decis Mak: Int J Soc Med Decis Mak. (2006) 26:565–74. doi: 10.1177/0272989X06295361 17099194 PMC2577036

[27] DeLong ER DeLong DM Clarke-Pearson DL . Comparing the areas under two or more correlated receiver operating characteristic curves: a nonparametric approach. Biometrics. (1988) 44:837–47. doi: 10.2307/2531595 3203132

[28] McInnes L Healy J Melville J . UMAP: uniform manifold approximation and projection for dimension reduction. (2020). doi: 10.48550/arXiv.1802.03426

[29] Selvaraju RR Cogswell M Das A Vedantam R Parikh D Batra D . Grad-CAM: visual explanations from deep networks via gradient-based localization. Int J Comput Vision. (2020) 128:336–59. doi: 10.1007/s11263-019-01228-7 30311153

[30] Bae S Choi YS Ahn SS Chang JH Kang S-G Kim EH . Radiomic MRI phenotyping of glioblastoma: improving survival prediction. Radiology. (2018) 289:797–806. doi: 10.1148/radiol.2018180200 30277442

[31] Nie D Zhang H Adeli E Liu L Shen D . 3D deep learning for multi-modal imaging-guided survival time prediction of brain tumor patients. Med Img Comput Comput-assist Interv: MICCAI Int Conf Med Img Comput Comput-Assist Interv. (2016) 9901:212–20. doi: 10.1007/978-3-319-46723-8_25 28149967 PMC5278791

[32] Yoon HG Cheon W Jeong SW Kim HS Kim K Nam H . Multi-parametric deep learning model for prediction of overall survival after postoperative concurrent chemoradiotherapy in glioblastoma patients. Cancers. (2020) 12:2284. doi: 10.3390/cancers12082284 32823939 PMC7465791

[33] Li Z-C Yan J Zhang S Liang C Lv X Zou Y . Glioma survival prediction from whole-brain MRI without tumor segmentation using deep attention network: a multicenter study. Eur Radio. (2022) 32:5719–29. doi: 10.1007/s00330-022-08640-7 35278123

[34] Luckett PH Olufawo M Lamichhane B Park KY Dierker D Verastegui GT . Predicting survival in glioblastoma with multimodal neuroimaging and machine learning. J Neuro-Oncol. (2023) 164:309–20. doi: 10.1007/s11060-023-04439-8 37668941 PMC10522528

[35] Akbari H Bakas S Sako C Fathi Kazerooni A Villanueva-Meyer J Garcia JA . Machine learning-based prognostic subgrouping of glioblastoma: a multicenter study. Neuro-Oncol. (2025) 27:1102–15. doi: 10.1093/neuonc/noae260 39665363 PMC12083074

[36] Soda Y Myskiw C Rommel A Verma IM . Mechanisms of neovascularization and resistance to anti-angiogenic therapies in glioblastoma multiforme. J Mol Med. (2013) 91:439–48. doi: 10.1007/s00109-013-1019-z 23512266 PMC3665343

[37] Rathore S Akbari H Doshi J Shukla G Rozycki M Bilello M . Radiomic signature of infiltration in peritumoral edema predicts subsequent recurrence in glioblastoma: implications for personalized radiotherapy planning. J Med Imaging. (2018) 5:21219. doi: 10.1117/1.JMI.5.2.021219 29531967 PMC5831697

[38] Prasanna P Patel J Partovi S Madabhushi A Tiwari P . Radiomic features from the peritumoral brain parenchyma on treatment-naïve multi-parametric MR imaging predict long versus short-term survival in glioblastoma multiforme: preliminary findings. Eur Radio. (2017) 27:4188–97. doi: 10.1007/s00330-016-4637-3 27778090 PMC5403632

[39] Dextraze K Saha A Kim D Narang S Lehrer M Rao A . Spatial habitats from multiparametric MR imaging are associated with signaling pathway activities and survival in glioblastoma. Oncotarget. (2017) 8:112992–3001. doi: 10.18632/oncotarget.22947 29348883 PMC5762568

[40] Choi SW Cho H-H Koo H Cho KR Nenning K-H Langs G . Multi-habitat radiomics unravels distinct phenotypic subtypes of glioblastoma with clinical and genomic significance. Cancers. (2020) 12:1707. doi: 10.3390/cancers12071707 32605068 PMC7408408

[41] Zhu Y Wang J Xue C Zhai X Xiao C Lu T . Deep learning and habitat radiomics for the prediction of glioma pathology using multiparametric MRI: a multicenter study. Acad Radiol. (2025) 32:963–75. doi: 10.1016/j.acra.2024.09.021 39322536

[42] Fathi Kazerooni A Akbari H Hu X Bommineni V Grigoriadis D Toorens E . The radiogenomic and spatiogenomic landscapes of glioblastoma and their relationship to oncogenic drivers. Commun Med. (2025) 5:55. doi: 10.1038/s43856-025-00767-0 40025245 PMC11873127

[43] Sarkaria JN Hu LS Parney IF Pafundi DH Brinkmann DH Laack NN . Is the blood-brain barrier really disrupted in all glioblastomas? A critical assessment of existing clinical data. Neuro-Oncol. (2018) 20:184–91. doi: 10.1093/neuonc/nox175 29016900 PMC5777482

[44] Curran WJ Scott CB Horton J Nelson JS Weinstein AS Fischbach AJ . Recursive partitioning analysis of prognostic factors in three radiation therapy oncology group Malignant glioma trials. J Natl Cancer Inst. (1993) 85:704–10. doi: 10.1093/jnci/85.9.704 8478956

[45] Chrysochoou D Gandhi DB Adib S Familiar AM Vunnava B Varshochi S . AI-powered segmentation and prognosis with missing MRI in pediatric brain tumors. NPJ Precis Oncol. (2026) 10:63. doi: 10.1038/s41698-025-01269-x 41530498 PMC12894653

[46] Chen M Zhang M Yin L Ma L Ding R Zheng T . Medical image foundation models in assisting diagnosis of brain tumors: a pilot study. Eur Radio. (2024) 34:6667–79. doi: 10.1007/s00330-024-10728-1 38627290

[47] Tak D Garomsa BA Zapaishchykova A Chaunzwa TL Climent Pardo JC Ye Z . A generalizable foundation model for analysis of human brain MRI. Nat Neurosci. (2026) 29:945–56. doi: 10.1038/s41593-026-02202-6 41644653 PMC13061609

