# Supplementary material for: Deep learning-based cross-attention fusion of multimodal MRI for survival prediction and risk stratification in IDH-wildtype glioblastoma: a multicenter study
Source: Front Oncol. 2026 Aug 24;16:1867582. doi: 10.3389/fonc.2026.1867582 (PMC13547015; doi:10.3389/fonc.2026.1867582)
Supplement: Supplementary file 1 [file DataSheet1.pdf]

Supplementary Material

Supplementary Figure S1. Heatmap of DeLong's test P values for pairwise model comparisons.

Supplementary Figure S2. Time-dependent ROC curves for all six deep learning models.

Supplementary Figure S3. Kaplan-Meier survival curves for all six deep learning models.

Supplementary Figure S4. UMAP dimensionality reduction visualization of the 1536-dimensional feature representations from the combined CAF model.

Supplementary Figure S5. Exploratory forest plot of C-indices (95% bootstrap CIs) for the combined CAF model across clinically relevant subgroups.

Supplementary Figure S6. Calibration curves for all six deep learning models at 12, 18, and 24-month time points.

Supplementary Figure S7. Kaplan-Meier survival curves of the combined CAF model in two external cohorts under a local-fit, external-inference setting.

Supplementary Table S1. Segmentation accuracy (Dice and 95% Hausdorff distance) of the nnU-Net models, evaluated by 5-fold cross-validation against BraTS expert ground truth.

Supplementary Table S2. Total and trainable parameter counts for each survival architecture.

Supplementary Table S3. Hyperparameters and training configurations across the three stages of model development.

Supplementary Table S4. Fold-specific versus global pretraining sensitivity analysis for the cross-attention fusion model.

Supplementary Table S5. Mask-perturbation sensitivity analysis for the cross-attention fusion model: out-of-fold C-index under whole-tumor erosion/dilation.

Supplementary Table S6. Exploratory FLAIR sub-analysis in the FLAIR-available subcohort (Local + TCGA, n = 324): CAF survival models with versus without FLAIR.

Supplementary Table S7. Proportional-hazards assumption assessment for the multivariable Cox model (n = 386): scaled Schoenfeld residual tests and hazard ratios.

Supplementary Table S8. Local-cohort sensitivity analysis of the deep learning risk score after additional adjustment for extent of resection and Karnofsky Performance Status.

Supplementary Table S9. Pre-treatment sensitivity analysis of the deep learning risk score with versus without adjuvant therapy.

Supplementary Table S10. Sensitivity analysis for missing MGMT promoter methylation status.

Supplementary Table S11. Quantitative calibration metrics at 12, 18, and 24 months for the combined CAF model and the six deep-learning-only models.

Supplementary Table S12. Diagnostic performance for predicting overall survival at 12, 18, and 24 months (Youden's optimal cutoff).

Supplementary Table S13. Integrated Brier Score (IBS) over 0-24 months for all models.

Supplementary Table S14. Leave-one-cohort-out (LOCO) external validation of the combined CAF model.

Supplementary Table S15. Performance of dual-modality combinations compared with single-modality and tri-modality models.

Supplementary Table S16. External validation: Cox models trained exclusively on the internal cohort and tested on CGGA and TCGA-GBM.

Supplementary Algorithm S1. End-to-end training and evaluation pseudocode for the multimodal MRI survival framework (Stage-1 segmentation-supervised pretraining, Stage-2 survival fine-tuning with 5-fold cross-validation, and Stage-3 Cox integration).

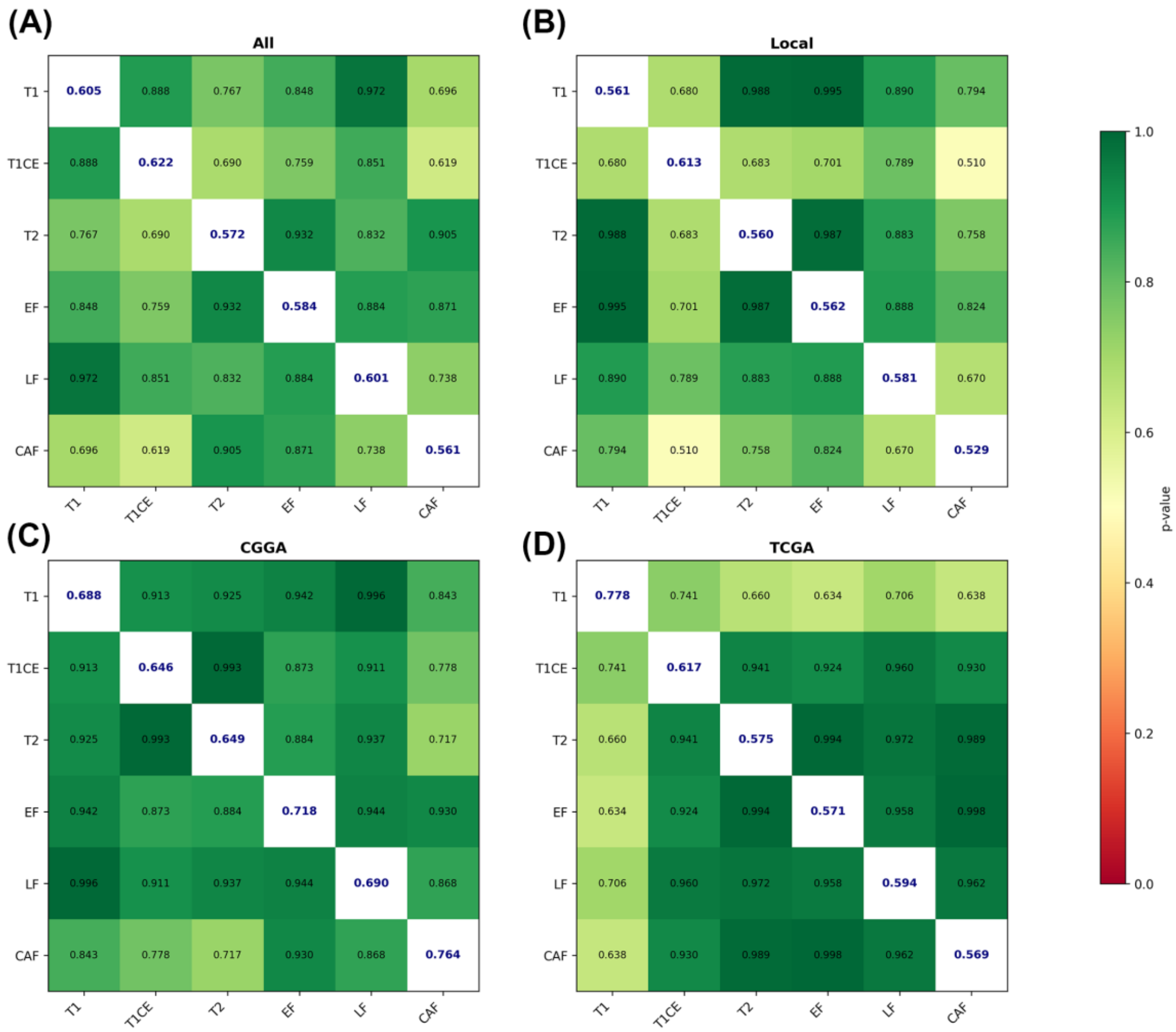

Supplementary Figure S1. Heatmap of DeLong's test *P* values for pairwise model comparisons. The layout is 2 rows × 2 columns, partitioned into four subplots based on evaluation scope: **(A)** all sample pooled (All), **(B)** institutional cohort (Local), **(C)** CGGA, and **(D)** TCGA. Each subplot presents a 6 × 6 heatmap where both rows and columns represent the six deep learning models (T1, T1CE, T2, early fusion EF, late fusion LF, cross-attention fusion CAF). Cell colors are encoded by a linear *P* value scale (0.0–1.0), with red indicating smaller *P* values (significant differences) and green indicating larger *P* values (non-significant). The lower and upper triangles are symmetrical. Cells along the main diagonal (white background, bold blue numbers) represent the model's self-reference values (lacking comparison significance). All off-diagonal cells represent the DeLong's test *P* value for differences in time-

dependent AUC between two given models, where $P > 0.05$ indicates the difference between models failed to reach statistical significance. **Abbreviations:** AUC, area under the curve; CAF, cross-attention fusion; EF, early fusion; LF, late fusion.

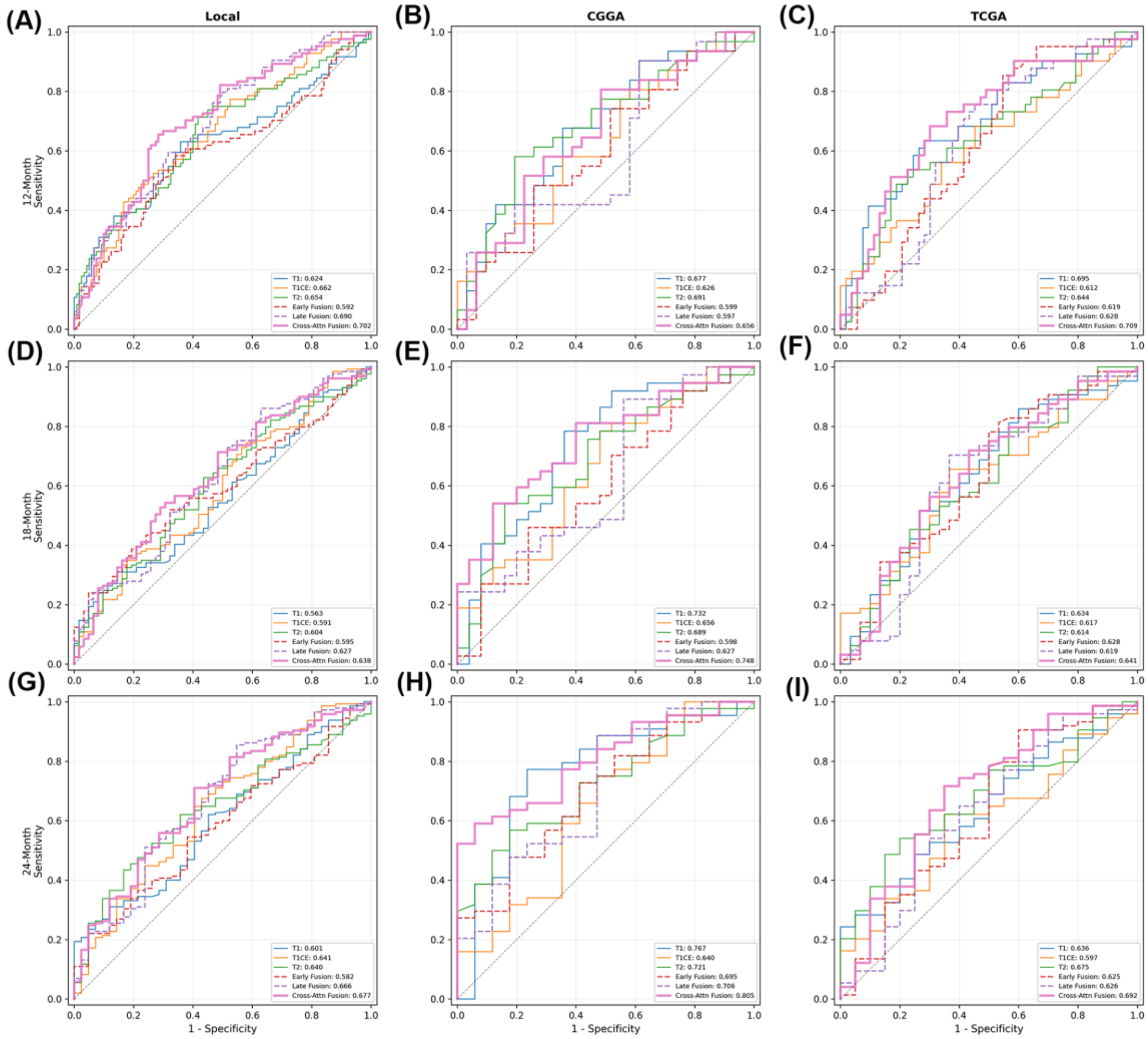

Supplementary Figure S2. Time-dependent ROC curves for all six deep learning models. This figure comprises 9 subplots arranged in a 3 rows × 3 columns layout. Rows correspond to the 12-, 18-, and 24-month time points (**(A–C)** 12 months, **(D–F)** 18 months, **(G–I)** 24 months), while columns correspond to the institutional, CGGA, and TCGA cohorts. Each subplot overlays the ROC curves of all six models (Model-T1, Model-T1CE, Model-T2, early fusion, late fusion, CAF). Single-modality models utilize thin solid lines, early/late fusion employ dashed lines, and CAF is highlighted with thick solid lines. AUC values for each model are annotated in the legend, and the black dashed line indicates the random classifier reference. AUCs are calculated based on binarized outcomes (event occurrence before the time point = positive). **Abbreviations:** AUC, area under the curve; CAF, cross-attention

fusion; ROC, receiver operating characteristic.

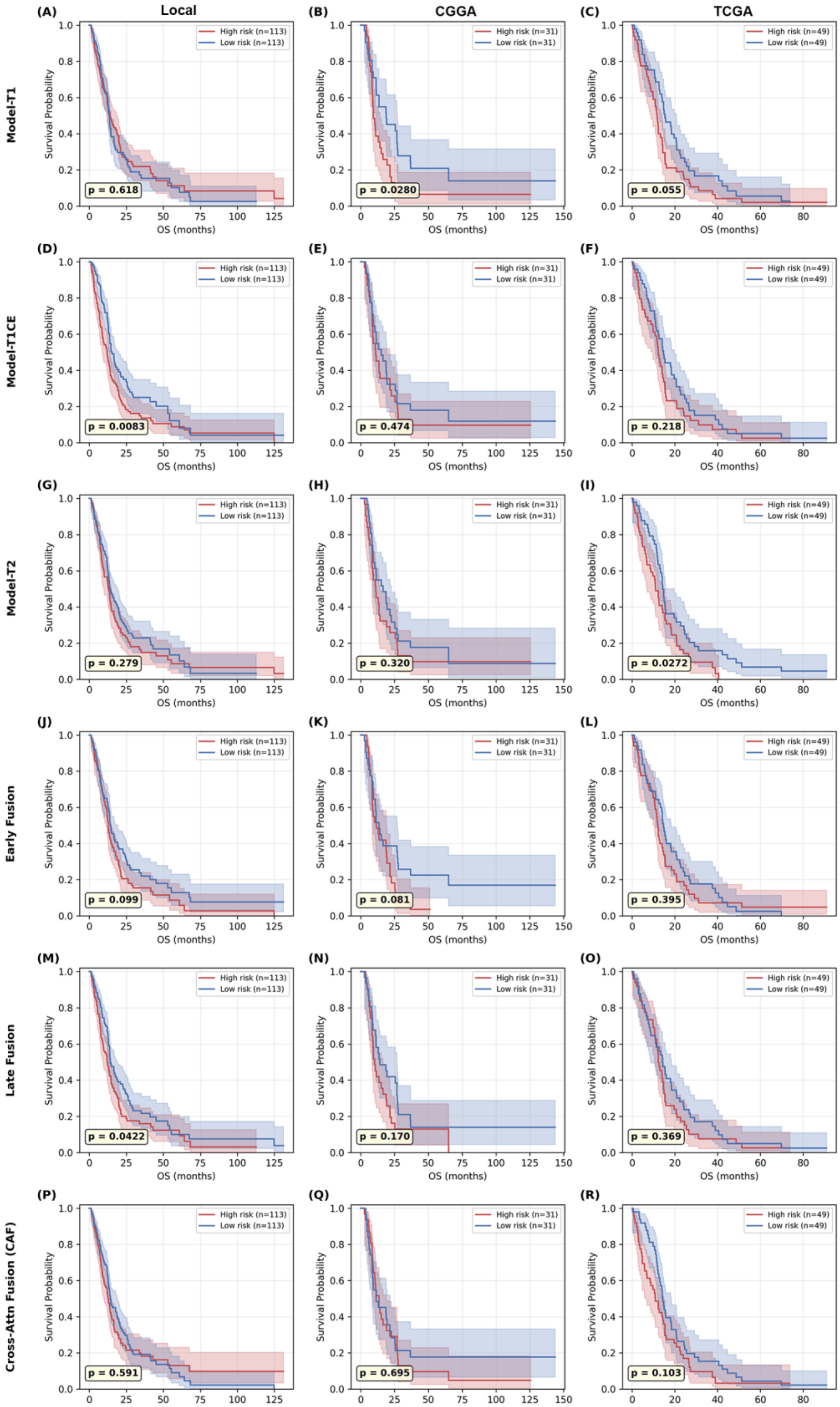

Local
CGGA
TCGA
Model-T1
Model-T1CE
Model-T2
Early Fusion
Late Fusion
Cross-Attn Fusion (CAF)
(A) p = 0.618
(B) p = 0.0280
(C) p = 0.055
(D) p = 0.0083
(E) p = 0.474
(F) p = 0.218
(G) p = 0.279
(H) p = 0.320
(I) p = 0.0272
(J) p = 0.099
(K) p = 0.081
(L) p = 0.395
(M) p = 0.0422
(N) p = 0.170
(O) p = 0.369
(P) p = 0.591
(Q) p = 0.695
(R) p = 0.103
High risk (n=113)
Low risk (n=113)
High risk (n=31)
Low risk (n=31)
High risk (n=49)
Low risk (n=49)
Survival Probability
OS (months)

Supplementary Figure S3. Kaplan-Meier survival curves for all six deep learning models. Comprising 18 subplots arranged in a 6 rows × 3 columns layout. Rows correspond to the six models (**(A–C)** Model-T1, **(D–F)** Model-T1CE, **(G–I)** Model-T2, **(J–L)** early fusion, **(M–O)** late fusion, **(P–R)** CAF), and columns correspond to the institutional, CGGA, and TCGA cohorts. Each subplot depicts survival curves with 95% confidence intervals for patients bifurcated into high (red) and low (blue) risk groups based on the cohort-specific median of the out-of-fold (cross-validated) risk scores within each respective cohort. Log-rank test *P* values are annotated within each subplot. **Abbreviations:** CAF, cross-attention fusion.

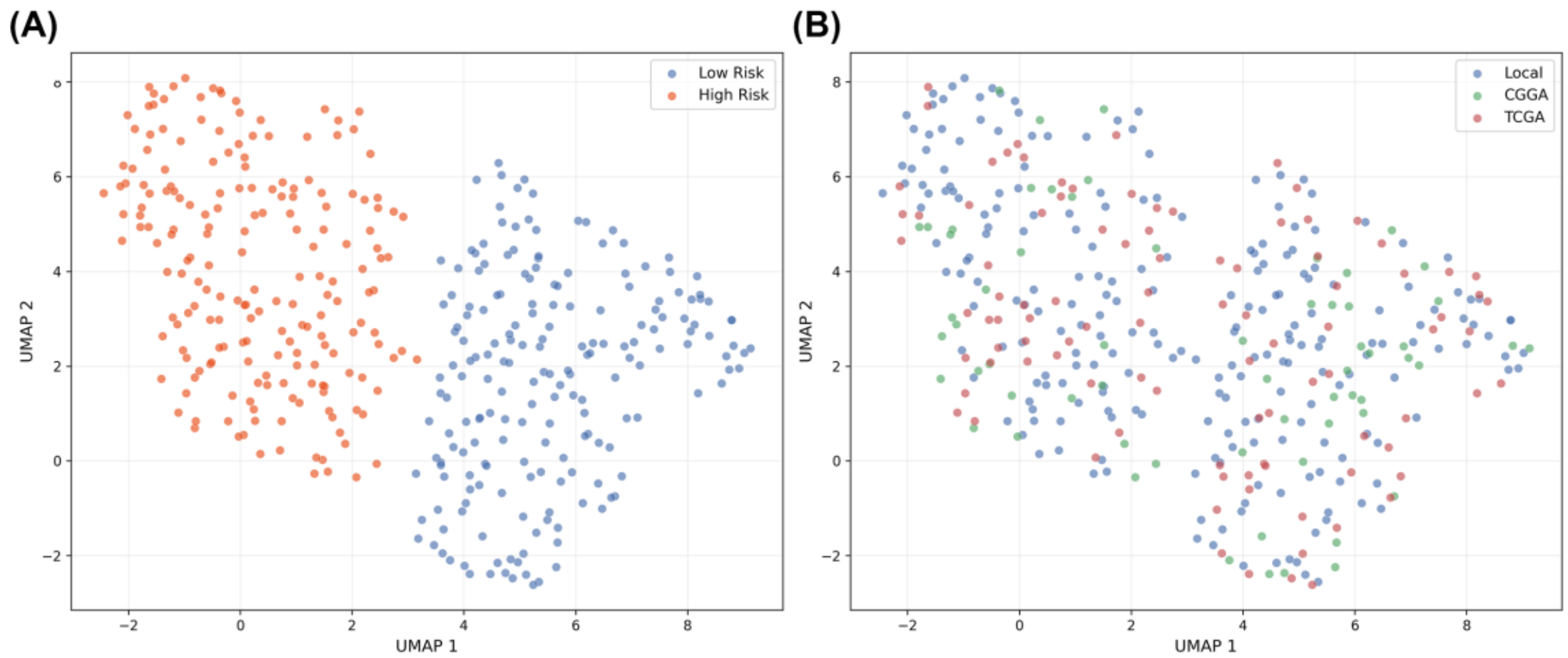

Supplementary Figure S4. UMAP dimensionality reduction visualization of the 1536-dimensional feature representations from the combined CAF model (A) Colored by risk group, where red indicates the high-risk group (higher than the median risk score in the training set) and blue indicates the low-risk group. The plot shows partial separation between the two groups within the two-dimensional UMAP space, suggesting that the learned features may capture risk-related signals. (B) Colored by cohort, utilizing three colors corresponding to the institutional, CGGA, and TCGA cohorts. Samples from the three centers appear well mixed within the feature space without prominent center-based clustering, which is qualitatively consistent with an absence of dominant batch effects. As an exploratory, non-linear visualization, UMAP cannot formally exclude batch effects. **Abbreviations:** UMAP, Uniform Manifold Approximation and Projection.

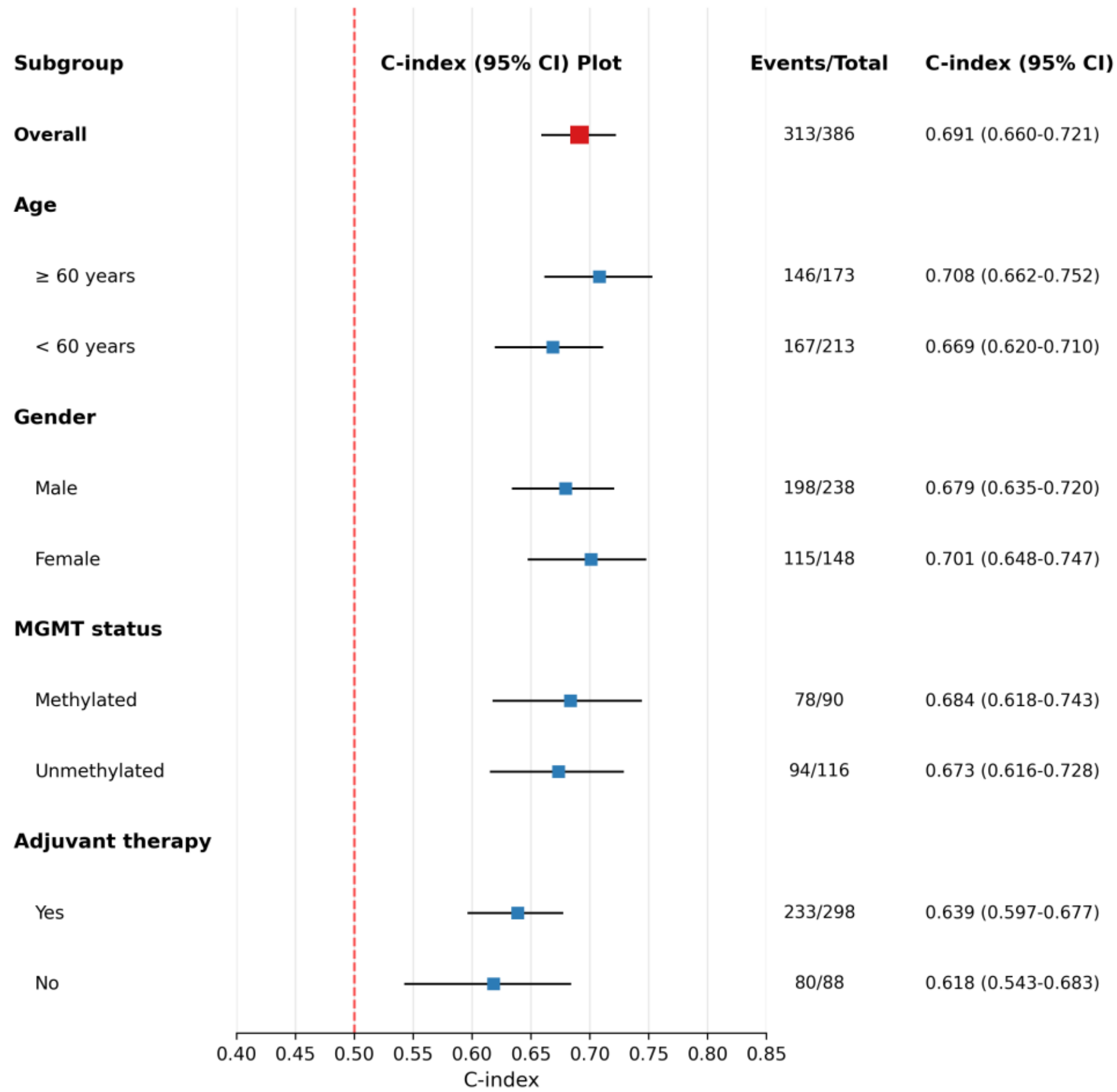

Supplementary Figure S5. Exploratory forest plot of C-indices (95% bootstrap CIs) for the combined CAF model across clinically relevant subgroups. The horizontal axis denotes C-index values. Each row corresponds to a subgroup (all patients, age ≥ 60 years vs. < 60 years, male vs. female, MGMT methylated vs. unmethylated, adjuvant therapy vs. no adjuvant therapy). Points represent the C-index point estimates, and horizontal whiskers indicate the 95% confidence intervals derived from 1000 bootstrap resampling iterations. The vertical reference line (C-index = 0.5) corresponds to the random prediction level. **Abbreviations:** CAF, cross-attention fusion; CI, confidence interval; MGMT, O^6-methylguanine-DNA methyltransferase.

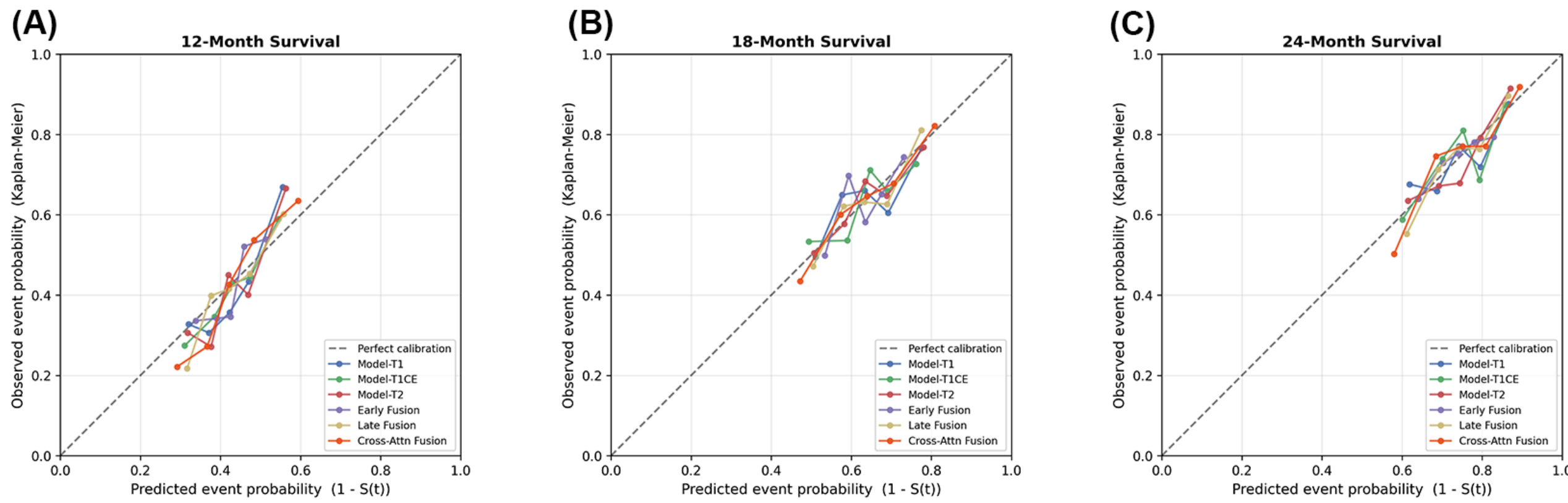

Supplementary Figure S6. Calibration curves for all six deep learning models at 12, 18, and 24-month time points. This layout consists of 1 row × 3 columns: **(A)** 12 months, **(B)** 18 months, and **(C)** 24 months. Each subplot overlays the calibration curves for all six deep learning models (Model-T1, Model-T1CE, Model-T2, early fusion, late fusion, cross-attention fusion). Individual models are connected by differently colored lines representing grouped data points. The black dashed line indicates the perfect calibration reference (y = x), where closer proximity to the reference signifies superior calibration performance. Predicted probabilities represent the out-of-fold survival-model estimates $1 - \hat{S}(t)$, and observed probabilities are the within-group Kaplan-Meier event fractions. The corresponding quantitative calibration metrics (O/E ratio, calibration slope, and calibration-in-the-large, with 95% CIs) for all models at 12, 18, and 24 months are tabulated in Supplementary Table S11. **Abbreviations:** CAF, cross-attention fusion.

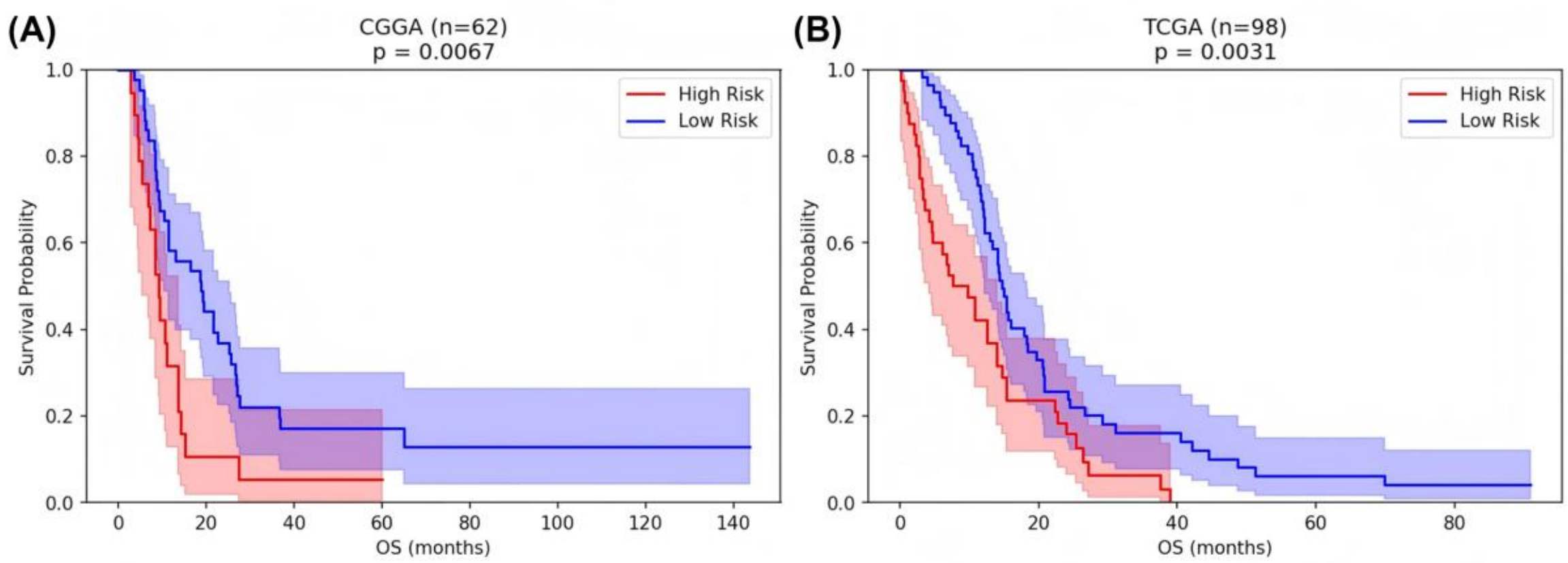

Supplementary Figure S7. Kaplan-Meier survival curves of the combined CAF model in two external cohorts under a local-fit, external-inference setting. This exploratory analysis maintained the fixed weights of the multicenter pretrained deep learning feature extractor, solely utilizing our institutional data (n = 226) to refit the Cox regression layer before applying it to two external inference cohorts. **(A)** CGGA cohort (n = 62), log-rank $P = 0.0067$; **(B)** TCGA cohort (n = 98), log-rank $P = 0.0031$. Both cohorts were stratified into binary high (red) and low (blue) risk groups using a single prespecified cutoff—the median risk score of the local (institutional) training cohort—applied unchanged to both external cohorts, with shaded areas denoting 95% confidence intervals. Both cohorts attained nominal statistical significance ($P < 0.01$). As this is an exploratory local-fit analysis—and because both external cohorts contributed to the multicenter feature-extractor training—these results are suggestive rather than confirmatory and should not be interpreted as a formal external validation. **Abbreviations:** CAF, cross-attention fusion.

Supplementary Table S1. Segmentation accuracy (Dice and 95% Hausdorff distance) of the nnU-Net models, evaluated by 5-fold cross-validation against BraTS expert ground truth.

Region	Dice 4-sequence (Local+TCGA)	Dice 3-sequence (CGGA)	HD95 (mm) 4-sequence (Local+TCGA)	HD95 (mm) 3-sequence (CGGA)
Whole tumor (labels 1+2+3)	0.938	0.910	3.56	4.64
Tumor core (labels 1+3)	0.920	0.915	2.96	3.06
Enhancing tumor (label 3)	0.878	0.873	2.38	2.58
Necrotic/non-enhancing core (label 1)	0.798	0.792	3.91	4.09
Peritumoral edema (label 2)	0.872	0.823	3.72	4.82

Values are means across the 5-fold cross-validation cases. The four-sequence model (T1, T1CE, T2, FLAIR) generated the masks for the Local and TCGA cohorts; the three-sequence model (T1, T1CE, T2) generated the masks for CGGA, which lacks FLAIR. The region of interest for the survival model was defined from the whole-tumor mask. Abbreviations: HD95, 95th-percentile Hausdorff distance.

Supplementary Table S2. Total and trainable parameter counts for each survival architecture.

Model	Encoders	Cross-attention	Total params (M)	Trainable params (M)
Model-T1	1	No	33.29	25.04
Model-T1CE	1	No	33.29	25.04
Model-T2	1	No	33.29	25.04
Early Fusion (EF)	1	No	33.34	25.04
Late Fusion (LF)	3	No	99.87	75.12
Cross-Attention Fusion (CAF)	3	Yes	106.18	81.43

Trainable counts reflect the two-stage policy (Stage-1 stem and layers 1–3 frozen; layer 4, the fusion modules, and the survival head fine-tuned). The three single-modality models are architecturally identical and therefore share the same counts. Cross-Attention Fusion adds only the cross-attention module (6.31 M, ≈6%) over the capacity-matched, attention-free Late Fusion control. The 3D ResNet-18 was selected as the unified backbone over deeper variants (ResNet-34/50/101) for three reasons: (i) with 386 pooled cases—considerably smaller than typical natural-image datasets—deeper networks (3D ResNet-50 ≈ 46 M parameters; ResNet-101 ≈ 85 M; versus ≈ 33 M here) carry substantially higher overfitting risk; (ii) the parameter count and memory overhead of 3D volumetric convolutions are substantially higher than their 2D counterparts, making deeper variants difficult to train stably with an adequate batch size on a single GPU at the 128 × 128 × 128 input size; and (iii) ResNet-18 has publicly available 3D medical pretrained weights (Med3D) that transfer directly into the two-stage paradigm. Abbreviations: M, million.

Supplementary Table S3. Hyperparameters and training configurations across the three stages of model development.

Stage	Parameter	Value
Stage 1: Segmentation Pre-training	Task	3D tumor segmentation (4-class)
	Backbone	3D ResNet-18 (encoder) + lightweight decoder
	Initial weights	Med3D pre-trained
	Loss function	Dice + Cross-Entropy (1:1)
	Optimizer	AdamW
	Learning rate	1e-3
	Weight decay	1e-4
	LR schedule	Cosine annealing (eta_min=1e-6)
	Epochs	100
	Batch size	2
	Input size	128x128x128
	Num classes	4 (BG, NCR, ED, ET)
Stage 2: Survival Fine-tuning	Loss function	Cox partial likelihood + L2 risk penalty (lambda=0.01)
	Optimizer	AdamW
	Base learning rate	1e-4
	Backbone layer4 LR	5e-5 (0.5x base)
	Fusion + head LR	1e-4 (1.0x base)
	Backbone freeze	stem + layer1-3 frozen
	Weight decay	1e-3
	LR schedule	Warmup (10 epochs) + Cosine annealing
	Max epochs	100
	Early stopping	Patience=30 (based on val C-index)
	Batch size	4
	Dropout (SurvivalHead)	0.5
	Dropout (CrossAttention)	0.1
	Cross-attention d_model	512
	Cross-attention n_heads	4

	Data augmentation	Random flip, rotation (+-15 deg), intensity shift (+-10%)
	CV strategy	5-fold stratified (OS_Status x Cohort)
Stage 3: Clinical Cox Integration	Model	CoxPH (lifelines)
	Penalizer (L2)	0.1
	Features	Age_std, Gender, MGMT_Status, MGMT_missing, Adjuvant_Therapy, dl_risk_std
	DL risk standardization	Per-fold z-score normalization
	CV strategy	Same 5-fold splits as Stage 2

Abbreviations: BG, background; CV, cross-validation; ED, peritumoral edema; ET, enhancing tumor; LR, learning rate; NCR, necrotic core; OS, overall survival.

Supplementary Table S4. Fold-specific versus global pretraining sensitivity analysis for the cross-attention fusion model.

(A) Out-of-fold discrimination (C-index) under each pretraining scheme

Pretraining scheme	Pooled	Local	CGGA	TCGA
Global (all 386 patients; manuscript)	0.629	0.623	0.619	0.648
Fold-specific (nested)	0.629	0.629	0.632	0.610

Paired-bootstrap difference (fold-specific − global), pooled C-index: +0.0001 (95% CI −0.038 to +0.036; P = 0.485 for fold-specific < global; 1,000 resamples), indicating no detectable optimistic bias from global pretraining.

(B) Per-fold segmentation whole-tumor Dice for the fold-specific encoders

Fold	T1	T1CE	T2
1	0.809	0.813	0.875
2	0.804	0.823	0.864
3	0.830	0.826	0.886
4	0.812	0.815	0.868
5	0.802	0.813	0.863
Mean	0.811	0.818	0.871

In the global scheme, the segmentation encoders were pretrained once on all 386 patients (segmentation supervision only; no survival labels) and loaded into every cross-validation fold. In the fold-specific scheme, the encoders for each fold were pretrained only on that fold's survival-training cases, so no validation-fold image was seen at any stage. C-indices are pooled out-of-fold estimates using the same per-fold standardization as the main analysis. Abbreviations: CAF, cross-attention fusion; CI, confidence interval.

Supplementary Table S5. Mask-perturbation sensitivity analysis for the cross-attention fusion model: out-of-fold C-index under whole-tumor erosion/dilation.

Condition	Pooled	Local	CGGA	TCGA	ΔC-index vs baseline (95% CI)
Baseline (unperturbed)	0.629	0.623	0.619	0.648	— (reference)
Erosion, 1 voxel	0.622	0.613	0.616	0.651	−0.007 (−0.016 to +0.001)
Dilation, 1 voxel	0.628	0.628	0.608	0.642	−0.001 (−0.010 to +0.008)
Erosion, 3 voxels	0.603	0.595	0.608	0.619	−0.026 (−0.047 to −0.004)
Dilation, 3 voxels	0.609	0.617	0.597	0.605	−0.020 (−0.036 to −0.002)
Erosion, 5 voxels	0.579	0.567	0.589	0.599	−0.050 (−0.082 to −0.021)
Dilation, 5 voxels	0.598	0.606	0.589	0.588	−0.030 (−0.051 to −0.008)

The whole-tumor mask was eroded or dilated by the indicated number of voxels, the 128^3 region of interest was regenerated with identical cropping logic, and the already-trained cross-attention fusion models were re-evaluated without retraining. C-indices are pooled and per-cohort out-of-fold estimates. The difference versus baseline and its 95% CI were obtained from 1,000 paired bootstrap resamples; intervals excluding zero indicate a significant change. Abbreviations: CI, confidence interval.

Supplementary Table S6. Exploratory FLAIR sub-analysis in the FLAIR-available subcohort (Local + TCGA, n = 324): CAF survival models with versus without FLAIR.

Model	C-index (standardized)	C-index (raw)	Local C-index	TCGA C-index	Mean time-dependent AUC
3-sequence CAF {T1, T1CE, T2}	0.637	0.632	0.647	0.623	0.701
4-sequence CAF {T1, T1CE, T2, FLAIR}	0.616	0.569	0.622	0.602	0.570

Both arms used identical stratified 5-fold splits, isolating the effect of FLAIR. The standardized C-index follows the main-analysis per-fold standardization and serves as the primary metric; under this metric the paired-bootstrap difference (4-sequence − 3-sequence; 1,000 resamples) was −0.021 (95% CI −0.057 to +0.014; P = 0.13), indicating no significant difference. For completeness, the corresponding difference under raw (unstandardized) pooling—which deflates the absolute cross-cohort C-index—was −0.065 (95% CI −0.117 to −0.010; P = 0.011). Under either pooling scheme, adding FLAIR did not improve discrimination. This exploratory result is confounded by the FLAIR stream lacking a dedicated pretrained encoder (initialized from T2) and the smaller subcohort, and should not be read as evidence that FLAIR is uninformative. Abbreviations: AUC, area under the curve; CAF, cross-attention fusion; CI, confidence interval; FLAIR, fluid-attenuated inversion recovery.

Supplementary Table S7. Proportional-hazards assumption assessment for the multivariable Cox model (n = 386): scaled Schoenfeld residual tests and hazard ratios.

(A) Primary multivariable Cox model

Covariate	Schoenfeld χ^2 (rank)	P (rank)	P (KM)	HR (95% CI)
Age (per SD)	1.16	0.281	0.233	1.26 (1.12–1.41)
Sex	0.82	0.364	0.373	1.16 (0.93–1.44)
MGMT status	0.70	0.403	0.404	0.95 (0.72–1.25)
MGMT-missing indicator	0.01	0.931	0.852	0.89 (0.70–1.14)
Adjuvant therapy	30.88	< 0.001	< 0.001	0.67 (0.59–0.77)
DL risk score (per SD)	5.26	0.022	0.022	1.41 (1.26–1.57)

(B) Cohort-stratified Cox model (separate baseline hazard per cohort)

Covariate	Schoenfeld χ^2 (rank)	P (rank)	HR (95% CI)
Age (per SD)	0.45	0.504	1.23 (1.10–1.38)
Sex	0.70	0.403	1.18 (0.94–1.48)
MGMT status	1.90	0.168	0.94 (0.71–1.24)
MGMT-missing indicator	1.47	0.225	0.88 (0.68–1.14)
Adjuvant therapy	3.39	0.066	0.63 (0.55–0.73)
DL risk score (per SD)	0.38	0.538	1.42 (1.27–1.59)

Scaled Schoenfeld residual tests; rank and Kaplan–Meier (KM) time transforms gave concordant results. Stratifying the baseline hazard by cohort removed all evidence of non-proportionality (all $P > 0.05$) with essentially unchanged hazard ratios. Abbreviations: CI, confidence interval; DL, deep learning; HR, hazard ratio; KM, Kaplan–Meier; MGMT, O^6-methylguanine-DNA methyltransferase; SD, standard deviation.

Supplementary Table S8. Local-cohort sensitivity analysis of the deep learning risk score after additional adjustment for extent of resection and Karnofsky Performance Status.

Model	Additional covariate(s)	DL risk score HR (95% CI)	P	Model C-index
Base	—	1.24 (1.07–1.43)	0.005	0.677
Base + KPS	KPS	1.24 (1.07–1.43)	0.004	0.677
Base + EOR	EOR	1.19 (1.03–1.39)	0.021	0.706
Base + KPS + EOR	KPS + EOR	1.19 (1.03–1.39)	0.021	0.706

Analysis restricted to the institutional cohort (n = 226; 169 events). All models adjusted for age, sex, MGMT status, and adjuvant therapy; DL risk score HR expressed per 1 SD. In the fully adjusted model, GTR was independently protective (HR 0.52, $P < 0.001$) and KPS ≥ 70 was not independently significant (HR 0.92, $P = 0.61$); a likelihood-ratio test confirmed significant incremental value of EOR + KPS ($\chi^2 = 16.70$, df = 2, $P < 0.001$). Near-identical Base and Base + KPS values reflect the lack of independent prognostic value of KPS. Abbreviations: CI, confidence interval; DL, deep learning; EOR, extent of resection; HR, hazard ratio; KPS, Karnofsky Performance Status; SD, standard deviation.

Supplementary Table S9. Pre-treatment sensitivity analysis of the deep learning risk score with versus without adjuvant therapy.

(A) Covariate hazard ratios (cohort-stratified Cox model)

Covariate	Full model (with adjuvant) HR (95% CI)	P	Preoperative model (no adjuvant) HR (95% CI)	P
Age (per 1 SD)	1.23 (1.10–1.38)	< 0.001	1.28 (1.14–1.44)	< 0.001
Sex (male vs female)	1.18 (0.94–1.48)	0.15	1.13 (0.90–1.41)	0.29
MGMT (methylated)	0.94 (0.71–1.24)	0.65	0.93 (0.70–1.23)	0.60
MGMT missing	0.88 (0.68–1.14)	0.33	0.83 (0.64–1.07)	0.14
Adjuvant therapy (yes vs no)	0.63 (0.55–0.73)	< 0.001	—	—
DL risk score (per 1 SD)	1.41 (1.26–1.57)	< 0.001	1.39 (1.25–1.56)	< 0.001

(B) Discrimination (out-of-fold C-index)

Model	Pooled	Local	CGGA	TCGA
Clinical only (preoperative, no adjuvant)	0.579	0.564	0.658	0.569
Deep-learning only	0.629	0.624	0.620	0.649
Combined (preoperative, no adjuvant)	0.642	0.631	0.672	0.645
Clinical only (full, with adjuvant)	0.656	0.654	0.695	0.647
Combined (full, with adjuvant)	0.691	0.688	0.716	0.689

Adjuvant therapy becomes known only after surgery; the full combined model is therefore a treatment-era model, whereas the pre-treatment model uses only preoperatively available covariates. Panel A: hazard ratios from the cohort-stratified

Cox model (penalizer = 0.1; n = 386); the proportional-hazards assumption held for the pre-treatment model (Schoenfeld test, all $P > 0.2$). Panel B: out-of-fold C-indices from the same five-fold cross-validation as the main analysis. Abbreviations: CI, confidence interval; DL, deep learning; HR, hazard ratio; MGMT, O^6-methylguanine-DNA methyltransferase; SD, standard deviation.

Supplementary Table S10. Sensitivity analysis for missing MGMT promoter methylation status.

(A) Complete-case multivariable Cox model (patients with documented MGMT status, n = 206; 172 events)

Covariate	HR (95% CI)	P
MGMT (methylated vs unmethylated)	0.99 (0.74–1.31)	0.92
Deep learning risk score (per 1 SD)	1.18 (1.02–1.36)	0.030
Age (per 1 SD)	1.32 (1.14–1.54)	< 0.001
Sex (male vs female)	1.22 (0.91–1.63)	0.18
Adjuvant therapy (yes vs no)	0.73 (0.61–0.88)	< 0.001

For comparison, the imputed full-cohort model (n = 386; Table 4) gave a similar MGMT estimate (HR 0.95, P = 0.72), suggesting that the non-significance of MGMT is unlikely to be primarily an artifact of the missing-indicator approach; this estimate should nonetheless be interpreted cautiously given the limited number of documented cases. Adding the deep learning risk score to the complete-case clinical model improved fit (likelihood-ratio test $\chi^2 = 5.49$, df = 1, P = 0.019; C-index 0.648 → 0.657).

(B) Comparison of patients with known versus unknown MGMT status

Variable	Known MGMT (n = 206)	Unknown MGMT (n = 180)	Test	P
Age, years (mean ± SD)	56.0 ± 13.4	57.3 ± 13.4	Welch t	0.34
Overall survival, months (mean ± SD)	18.6 ± 23.2	17.0 ± 15.0	Welch t	0.42
DL risk score (mean ± SD)	−0.23 ± 1.34	−0.03 ± 1.23	Welch t	0.13
Male sex, n	119	119	χ^2	0.11
Death events, n (%)	172 (83.5)	141 (78.3)	χ^2	0.25
Cohort (CGGA / Local / TCGA), n	53 / 101 / 52	9 / 125 / 46	χ^2	< 0.001
Adjuvant therapy (0 / 1 / 2), n	39 / 47 / 117	49 / 24 / 107	χ^2	0.021

Panel A: multivariable Cox model (penalizer = 0.1) fit on the 206 patients with documented MGMT status, MGMT entered directly (no imputation). Panel B: MGMT missingness differed mainly by cohort and the associated adjuvant-therapy distribution, indicating it was driven by site-level data curation rather than by patient outcome. The imaging-derived score is a complementary dimension, not a replacement for MGMT testing. Abbreviations: CI, confidence interval; DL, deep learning; HR, hazard ratio; MGMT, O^6-methylguanine-DNA methyltransferase; SD, standard deviation.

Supplementary Table S11. Quantitative calibration metrics at 12, 18, and 24 months for the combined CAF model and the six deep-learning-only models.

Model	Time (months)	O/E ratio (95% CI)	Calibration slope (95% CI)	Calibration-in-the-large (95% CI)
Combined CAF (DL + clinical)	12	0.94 (0.83–1.04)	1.29 (0.96–1.61)	-0.07 (-0.23–0.08)
	18	0.97 (0.90–1.05)	0.92 (0.65–1.19)	-0.07 (-0.21–0.08)
	24	0.98 (0.93–1.04)	0.86 (0.56–1.16)	-0.06 (-0.21–0.10)
Model-T1 (DL-only)	12	0.98 (0.86–1.10)	1.49 (0.85–2.13)	-0.02 (-0.18–0.14)
	18	1.00 (0.93–1.07)	0.87 (0.34–1.40)	-0.01 (-0.15–0.13)
	24	1.00 (0.94–1.06)	0.99 (0.45–1.54)	-0.00 (-0.14–0.14)
Model-T1CE (DL-only)	12	0.97 (0.86–1.09)	1.41 (0.80–2.02)	-0.03 (-0.19–0.13)
	18	1.00 (0.91–1.08)	1.02 (0.51–1.53)	-0.00 (-0.14–0.14)
	24	1.00 (0.94–1.06)	0.95 (0.41–1.48)	-0.00 (-0.14–0.14)
Model-T2 (DL-only)	12	0.97 (0.87–1.09)	1.37 (0.75–1.98)	-0.03 (-0.18–0.13)
	18	0.99 (0.91–1.08)	0.91 (0.39–1.43)	-0.01 (-0.15–0.13)
	24	1.00 (0.94–1.06)	1.13 (0.60–1.65)	-0.00 (-0.14–0.13)
Early Fusion (DL-only)	12	0.98 (0.87–1.10)	1.21 (0.35–2.06)	-0.02 (-0.18–0.14)
	18	1.00 (0.92–1.08)	0.91 (0.20–1.63)	0.00 (-0.14–0.14)
	24	1.00 (0.94–1.07)	0.89 (0.20–1.57)	0.01 (-0.13–0.14)
Late Fusion (DL-only)	12	0.98 (0.86–1.09)	1.41 (0.82–2.00)	-0.03 (-0.18–0.13)

	18	1.00 (0.92–1.08)	0.96 (0.41–1.51)	-0.01 (-0.14–0.13)
	24	1.00 (0.94–1.06)	1.10 (0.53–1.67)	0.01 (-0.13–0.14)
	12	0.97 (0.86–1.07)	1.42 (0.92–1.92)	-0.03 (-0.19–0.13)
Cross-Attn Fusion (DL-only)	18	0.99 (0.92–1.07)	1.00 (0.58–1.43)	-0.01 (-0.15–0.13)
	24	1.00 (0.94–1.05)	1.18 (0.74–1.62)	0.00 (-0.14–0.14)

All metrics were computed from out-of-fold (cross-validated) Cox predictions. The observed/expected (O/E) ratio compares the cohort-level Kaplan–Meier observed event probability with the mean predicted event probability ($1 - \hat{S}(t)$); 95% confidence intervals (CIs) were obtained from 1,000 nonparametric bootstrap resamples. The calibration slope and calibration-in-the-large (calibration intercept) were estimated from leave-one-out jackknife pseudo-observations of the event probability on the complementary log-log scale, with robust (sandwich) 95% CIs. An O/E ratio of 1, a slope of 1, and an intercept of 0 indicate perfect calibration. Abbreviations: CAF, cross-attention fusion; CI, confidence interval; DL, deep learning; O/E, observed/expected.

Supplementary Table S12. Diagnostic performance for predicting overall survival at 12, 18, and 24 months (Youden's optimal cutoff).

Model	Cohort	Time	N	Events	AUC (95% CI)	Accuracy (95% CI)	Sensitivity (95% CI)	Specificity (95% CI)	PPV (95% CI)	NPV (95% CI)
Clinical Only	Internal	12-month	204	84	0.732 (0.659-0.798)	0.735 (0.672-0.794)	0.631 (0.527-0.729)	0.808 (0.726-0.868)	0.697 (0.579-0.782)	0.758 (0.681-0.829)
Clinical Only	CGGA	12-month	62	31	0.759 (0.623-0.871)	0.581 (0.452-0.710)	0.194 (0.061-0.333)	0.968 (0.896-1.000)	0.857 (0.500-1.000)	0.545 (0.408-0.673)
Clinical Only	TCGA	12-month	94	41	0.681 (0.555-0.778)	0.670 (0.574-0.755)	0.341 (0.205-0.477)	0.925 (0.836-0.982)	0.778 (0.550-0.947)	0.645 (0.538-0.747)
Clinical Only	Pooled	12-month	360	156	0.710 (0.657-0.761)	0.692 (0.647-0.739)	0.468 (0.394-0.547)	0.863 (0.819-0.909)	0.723 (0.636-0.809)	0.680 (0.624-0.732)
Clinical Only	Internal	18-month	191	129	0.660 (0.577-0.740)	0.623 (0.555-0.686)	0.519 (0.436-0.603)	0.839 (0.733-0.929)	0.870 (0.787-0.945)	0.456 (0.372-0.550)
Clinical Only	CGGA	18-month	62	37	0.762 (0.626-0.878)	0.548 (0.435-0.677)	0.243 (0.108-0.381)	1.000 (1.000-1.000)	1.000 (1.000-1.000)	0.472 (0.345-0.608)
Clinical Only	TCGA	18-month	94	64	0.688 (0.570-0.796)	0.489 (0.394-0.596)	0.266 (0.162-0.375)	0.967 (0.885-1.000)	0.944 (0.818-1.000)	0.382 (0.272-0.494)
Clinical Only	Pooled	18-month	347	230	0.685 (0.625-0.743)	0.573 (0.524-0.628)	0.404 (0.341-0.470)	0.906 (0.847-0.956)	0.894 (0.827-0.950)	0.436 (0.377-0.504)
Clinical Only	Internal	24-month	187	145	0.660 (0.571-0.743)	0.599 (0.535-0.663)	0.545 (0.468-0.624)	0.786 (0.658-0.905)	0.898 (0.833-0.954)	0.333 (0.242-0.430)
Clinical Only	CGGA	24-month	61	44	0.795 (0.667-0.900)	0.508 (0.377-0.639)	0.318 (0.190-0.465)	1.000 (1.000-1.000)	1.000 (1.000-1.000)	0.362 (0.234-0.500)
Clinical Only	TCGA	24-month	94	74	0.643 (0.507-0.772)	0.436 (0.340-0.532)	0.324 (0.222-0.429)	0.850 (0.667-1.000)	0.889 (0.750-1.000)	0.254 (0.156-0.357)
Clinical Only	Pooled	24-month	342	263	0.678 (0.618-0.739)	0.538 (0.488-0.591)	0.445 (0.387-0.502)	0.848 (0.762-0.920)	0.907 (0.855-0.953)	0.315 (0.256-0.378)
DL-only (CAF)	Internal	12-month	204	84	0.703 (0.630-0.774)	0.691 (0.627-0.755)	0.655 (0.550-0.759)	0.717 (0.637-0.798)	0.618 (0.517-0.716)	0.748 (0.667-0.829)
DL-only (CAF)	CGGA	12-month	62	31	0.658 (0.520-0.783)	0.629 (0.500-0.742)	0.548 (0.379-0.714)	0.710 (0.545-0.852)	0.654 (0.474-0.810)	0.611 (0.441-0.758)
DL-only (CAF)	TCGA	12-month	94	41	0.709 (0.594-0.815)	0.670 (0.574-0.766)	0.634 (0.477-0.778)	0.698 (0.566-0.818)	0.619 (0.467-0.750)	0.712 (0.582-0.836)
DL-only (CAF)	Pooled	12-month	360	156	0.697 (0.644-0.753)	0.675 (0.628-0.720)	0.628 (0.553-0.703)	0.711 (0.647-0.770)	0.624 (0.553-0.696)	0.714 (0.649-0.773)
DL-only	Internal	18-	191	129	0.639	0.581	0.512	0.726	0.795	0.417

(CAF)		month			(0.549-0.724)	(0.508-0.649)	(0.422-0.598)	(0.612-0.836)	(0.704-0.876)	(0.325-0.509)
DL-only (CAF)	CGGA	18-month	62	37	0.751 (0.619-0.864)	0.661 (0.532-0.774)	0.568 (0.400-0.722)	0.800 (0.619-0.931)	0.808 (0.652-0.939)	0.556 (0.382-0.706)
DL-only (CAF)	TCGA	18-month	94	64	0.641 (0.500-0.771)	0.574 (0.468-0.681)	0.516 (0.391-0.646)	0.700 (0.522-0.864)	0.786 (0.659-0.909)	0.404 (0.263-0.542)
DL-only (CAF)	Pooled	18-month	347	230	0.663 (0.604-0.726)	0.594 (0.542-0.646)	0.522 (0.454-0.584)	0.735 (0.652-0.820)	0.795 (0.726-0.857)	0.439 (0.367-0.514)
DL-only (CAF)	Internal	24-month	187	145	0.677 (0.586-0.763)	0.684 (0.615-0.749)	0.710 (0.632-0.785)	0.595 (0.450-0.735)	0.858 (0.793-0.915)	0.373 (0.264-0.492)
DL-only (CAF)	CGGA	24-month	61	44	0.803 (0.690-0.902)	0.738 (0.623-0.836)	0.773 (0.638-0.884)	0.647 (0.400-0.867)	0.850 (0.738-0.950)	0.524 (0.304-0.739)
DL-only (CAF)	TCGA	24-month	94	74	0.694 (0.540-0.830)	0.713 (0.617-0.798)	0.743 (0.637-0.842)	0.600 (0.333-0.818)	0.873 (0.785-0.952)	0.387 (0.208-0.562)
DL-only (CAF)	Pooled	24-month	342	263	0.708 (0.636-0.769)	0.702 (0.655-0.749)	0.730 (0.676-0.785)	0.608 (0.485-0.710)	0.861 (0.807-0.905)	0.403 (0.316-0.487)
Combined (CAF)	Internal	12-month	204	84	0.789 (0.720-0.847)	0.770 (0.716-0.824)	0.631 (0.530-0.730)	0.867 (0.798-0.924)	0.768 (0.667-0.859)	0.770 (0.695-0.839)
Combined (CAF)	CGGA	12-month	62	31	0.784 (0.645-0.889)	0.581 (0.468-0.694)	0.258 (0.103-0.419)	0.903 (0.781-1.000)	0.727 (0.428-1.000)	0.549 (0.412-0.680)
Combined (CAF)	TCGA	12-month	94	41	0.742 (0.623-0.837)	0.670 (0.574-0.755)	0.341 (0.194-0.487)	0.925 (0.840-0.982)	0.778 (0.550-0.950)	0.645 (0.538-0.746)
Combined (CAF)	Pooled	12-month	360	156	0.764 (0.718-0.812)	0.711 (0.667-0.756)	0.481 (0.405-0.557)	0.887 (0.844-0.930)	0.765 (0.684-0.848)	0.691 (0.634-0.743)
Combined (CAF)	Internal	18-month	191	129	0.706 (0.626-0.780)	0.654 (0.586-0.723)	0.589 (0.507-0.669)	0.790 (0.683-0.887)	0.854 (0.775-0.924)	0.480 (0.386-0.578)
Combined (CAF)	CGGA	18-month	62	37	0.834 (0.715-0.925)	0.629 (0.500-0.742)	0.405 (0.237-0.559)	0.960 (0.864-1.000)	0.938 (0.800-1.000)	0.522 (0.386-0.660)
Combined (CAF)	TCGA	18-month	94	64	0.712 (0.597-0.819)	0.532 (0.436-0.638)	0.375 (0.258-0.500)	0.867 (0.735-0.968)	0.857 (0.714-0.963)	0.394 (0.278-0.520)
Combined (CAF)	Pooled	18-month	347	230	0.730 (0.673-0.781)	0.617 (0.565-0.669)	0.500 (0.436-0.560)	0.846 (0.784-0.911)	0.865 (0.801-0.923)	0.463 (0.394-0.533)
Combined (CAF)	Internal	24-month	187	145	0.721 (0.642-0.800)	0.679 (0.610-0.743)	0.655 (0.580-0.734)	0.762 (0.628-0.886)	0.905 (0.845-0.955)	0.390 (0.289-0.494)
Combined (CAF)	CGGA	24-month	61	44	0.888 (0.796-0.960)	0.689 (0.574-0.787)	0.568 (0.428-0.696)	1.000 (1.000-1.000)	1.000 (1.000-1.000)	0.472 (0.316-0.622)
Combined (CAF)	TCGA	24-month	94	74	0.711 (0.574-0.828)	0.574 (0.468-0.681)	0.514 (0.400-0.625)	0.800 (0.611-0.950)	0.905 (0.820-0.977)	0.308 (0.184-0.429)

Combined (CAF)	Pooled	24-month	342	263	0.749 (0.686-0.804)	0.652 (0.602-0.705)	0.601 (0.542-0.663)	0.823 (0.729-0.904)	0.919 (0.873-0.957)	0.382 (0.309-0.455)

The optimal cutoff was determined by maximizing Youden's J index (sensitivity + specificity - 1) on the internal cohort and then applied to all cohorts without re-estimation. Patients censored before the time point with no observed event were excluded from the analysis at that time point. All metrics are presented as point estimates with 95% CIs obtained from 1,000 bootstrap resamples. Abbreviations: AUC, area under the receiver operating characteristic curve; CAF, cross-attention fusion; CI, confidence interval; DL, deep learning; NPV, negative predictive value; PPV, positive predictive value.

Supplementary Table S13. Integrated Brier Score (IBS) over 0-24 months for all models.

Model	Architecture	Pooled IBS (95% CI)	Internal IBS (95% CI)	CGGA IBS (95% CI)	TCGA IBS (95% CI)
KM Null Model	—	0.190	0.188	0.224	0.202
Clinical Only	—	0.171 (0.161-0.183)	0.171 (0.156-0.186)	0.193 (0.178-0.220)	0.184 (0.167-0.204)
DL-only (T1)	T1	0.184 (0.174-0.196)	0.185 (0.171-0.198)	0.210 (0.190-0.240)	0.194 (0.177-0.214)
DL-only (T1CE)	T1CE	0.183 (0.173-0.194)	0.181 (0.167-0.194)	0.213 (0.193-0.243)	0.197 (0.180-0.216)
DL-only (T2)	T2	0.182 (0.173-0.194)	0.182 (0.169-0.197)	0.211 (0.193-0.239)	0.193 (0.174-0.215)
DL-only (Early Fusion)	Early Fusion	0.188 (0.179-0.199)	0.187 (0.174-0.201)	0.224 (0.202-0.256)	0.197 (0.181-0.216)
DL-only (Late Fusion)	Late Fusion	0.182 (0.173-0.194)	0.178 (0.166-0.192)	0.215 (0.198-0.243)	0.199 (0.179-0.221)
DL-only (CAF)	CAF	0.178 (0.168-0.189)	0.178 (0.164-0.191)	0.202 (0.184-0.229)	0.190 (0.172-0.211)
Combined (T1)	T1	0.166 (0.155-0.178)	0.168 (0.152-0.184)	0.179 (0.163-0.205)	0.178 (0.159-0.200)
Combined (T1CE)	T1CE	0.166 (0.156-0.178)	0.165 (0.150-0.181)	0.187 (0.169-0.216)	0.181 (0.163-0.202)
Combined (T2)	T2	0.165 (0.155-0.177)	0.165 (0.150-0.181)	0.185 (0.168-0.213)	0.178 (0.159-0.199)
Combined (Early Fusion)	Early Fusion	0.167 (0.157-0.179)	0.167 (0.153-0.184)	0.186 (0.165-0.217)	0.179 (0.160-0.200)
Combined (Late Fusion)	Late Fusion	0.165 (0.154-0.177)	0.162 (0.147-0.179)	0.185 (0.169-0.213)	0.182 (0.162-0.206)
Combined (CAF)	CAF	0.161 (0.150-0.173)	0.162 (0.147-0.178)	0.177 (0.159-0.204)	0.172 (0.155-0.193)

The IBS integrates the squared error between predicted and observed survival probabilities over 0-24 months; lower values indicate better calibrated predictions. The Kaplan-Meier null model serves as a non-informative baseline. All IBS values are presented as point estimates with 95% CIs obtained from 1,000 bootstrap resamples. Censoring distribution was estimated from the full study cohort. Abbreviations: CAF, cross-attention fusion; CI, confidence interval; DL, deep

learning; IBS, integrated Brier score; KM, Kaplan-Meier.

Supplementary Table S14. Leave-one-cohort-out (LOCO) external validation of the combined CAF model.

Held-out cohort (n)	Model	C-index	Mean tdAUC (12/18/24 m)	KM log-rank P
Local (226)	Clinical-only	0.656	0.701	< 0.001
	DL-only	0.564	0.582	0.076
	Combined	0.671	0.720	< 0.001
CGGA (62)	Clinical-only	0.703	0.762	0.003
	DL-only	0.589	0.662	0.027
	Combined	0.708	0.792	< 0.001
TCGA (98)	Clinical-only	0.637	0.651	0.118
	DL-only	0.561	0.581	0.130
	Combined	0.632	0.650	0.007

The Clinical-only model used age, sex, MGMT status (with missing indicator), and adjuvant therapy; the Combined model additionally incorporated the standardized DL risk score. For KM analysis, the high-/low-risk threshold was the median risk score of the training cohorts applied to the held-out cohort. Abbreviations: AUC, area under the receiver operating characteristic curve; CAF, cross-attention fusion; CGGA, Chinese Glioma Genome Atlas; C-index, concordance index; DL, deep learning; KM, Kaplan–Meier; LOCO, leave-one-cohort-out; TCGA, The Cancer Genome Atlas; tdAUC, time-dependent AUC.

Supplementary Table S15. Performance of dual-modality combinations compared with single-modality and tri-modality models.

Model	Modality Combination	Cross-Validation C-index (Mean ± SD)	Pooled C-index (95% CI)	Internal C-index (95% CI)	CGGA C-index (95% CI)	TCGA C-index (95% CI)
Combined (T1 + T1CE)	T1 + T1CE	0.684 ± 0.023	0.683 (0.650-0.714)	0.682 (0.636-0.725)	0.710 (0.639-0.771)	0.666 (0.590-0.730)
Combined (T1 + T2)	T1 + T2	0.683 ± 0.029	0.680 (0.650-0.710)	0.677 (0.631-0.717)	0.727 (0.657-0.788)	0.665 (0.592-0.730)
Combined (T1CE + T2)	T1CE + T2	0.682 ± 0.024	0.682 (0.650-0.712)	0.687 (0.640-0.726)	0.700 (0.628-0.765)	0.657 (0.578-0.716)
DL-only (T1 + T1CE)	T1 + T1CE	0.616 ± 0.027	0.612 (0.579-0.648)	0.609 (0.560-0.660)	0.614 (0.527-0.697)	0.619 (0.552-0.681)
DL-only (T1 + T2)	T1 + T2	0.608 ± 0.020	0.607 (0.571-0.641)	0.584 (0.534-0.634)	0.639 (0.562-0.705)	0.633 (0.557-0.697)
DL-only (T1CE + T2)	T1CE + T2	0.624 ± 0.011	0.622 (0.588-0.660)	0.622 (0.572-0.673)	0.614 (0.531-0.688)	0.626 (0.556-0.693)

Dual-modality DL risk scores were obtained by averaging the standardized risk scores from two single-modality models. Cross-validation C-index is presented as mean ± SD across 5 folds; pooled and per-cohort C-indices are presented as point estimates with 95% CIs from 1,000 bootstrap resamples. Abbreviations: CI, confidence interval; DL, deep learning; SD, standard deviation.

Supplementary Table S16. External validation: Cox models trained exclusively on the internal cohort and tested on CGGA and TCGA-GBM.

Model	Underlying DL Architecture	Internal CV C-index (95% CI)	CGGA External C-index (95% CI)	TCGA External C-index (95% CI)	External Pooled C-index (95% CI)
Clinical Only	T1	0.643 (0.599-0.687)	0.690 (0.607-0.764)	0.618 (0.534-0.682)	0.643 (0.586-0.694)
DL-only (T1)	T1	0.538 (0.493-0.590)	0.593 (0.502-0.685)	0.606 (0.540-0.674)	0.600 (0.547-0.651)
Combined (T1)	T1	0.650 (0.606-0.694)	0.714 (0.643-0.782)	0.628 (0.542-0.692)	0.661 (0.611-0.709)
Clinical Only	T1CE	0.643 (0.599-0.687)	0.690 (0.607-0.764)	0.618 (0.534-0.682)	0.643 (0.586-0.694)
DL-only (T1CE)	T1CE	0.593 (0.542-0.642)	0.569 (0.483-0.645)	0.563 (0.503-0.623)	0.566 (0.518-0.615)
Combined (T1CE)	T1CE	0.659 (0.613-0.706)	0.704 (0.629-0.773)	0.628 (0.545-0.690)	0.654 (0.596-0.703)
Clinical Only	T2	0.643 (0.599-0.687)	0.690 (0.607-0.764)	0.618 (0.534-0.682)	0.643 (0.586-0.694)
DL-only (T2)	T2	0.547 (0.501-0.600)	0.568 (0.485-0.647)	0.591 (0.525-0.650)	0.584 (0.533-0.632)
Combined (T2)	T2	0.645 (0.604-0.689)	0.697 (0.621-0.770)	0.640 (0.556-0.702)	0.665 (0.613-0.711)
Clinical Only	Early Fusion	0.643 (0.599-0.687)	0.690 (0.607-0.764)	0.618 (0.534-0.682)	0.643 (0.586-0.694)
DL-only (Early Fusion)	Early Fusion	0.533 (0.485-0.580)	0.555 (0.455-0.651)	0.584 (0.511-0.652)	0.558 (0.502-0.613)
Combined (Early Fusion)	Early Fusion	0.640 (0.596-0.683)	0.715 (0.620-0.792)	0.644 (0.567-0.704)	0.667 (0.614-0.718)
Clinical Only	Late Fusion	0.643 (0.599-0.687)	0.690 (0.607-0.764)	0.618 (0.534-0.682)	0.643 (0.586-0.694)
DL-only (Late Fusion)	Late Fusion	0.580 (0.534-0.626)	0.537 (0.457-0.619)	0.543 (0.471-0.613)	0.541 (0.490-0.596)
Combined (Late Fusion)	Late Fusion	0.657 (0.617-0.698)	0.680 (0.593-0.758)	0.621 (0.542-0.683)	0.643 (0.591-0.693)
Clinical Only	CAF	0.643 (0.599-0.687)	0.690 (0.607-0.764)	0.618 (0.534-0.682)	0.643 (0.586-0.694)
DL-only (CAF)	CAF	0.561 (0.512-0.613)	0.532 (0.445-0.618)	0.574 (0.504-0.645)	0.562 (0.508-0.616)
Combined (CAF)	CAF	0.656 (0.614-0.702)	0.664 (0.579-0.743)	0.626 (0.539-0.691)	0.645 (0.589-0.693)

The Cox model was fit on the internal cohort using 5-fold CV; for each fold, predictions on CGGA and TCGA-GBM were averaged across folds (ensemble) to obtain external risk scores. C-indices are presented as point estimates with

95% CIs from 1,000 bootstrap resamples. Abbreviations: CAF, cross-attention fusion; CI, confidence interval; CV, cross-validation; DL, deep learning.

Supplementary Algorithm S1. End-to-end training and evaluation pseudocode for the multimodal MRI survival framework.

Pseudocode for the complete pipeline. A single global seed (42) is fixed for Python, NumPy, and PyTorch (with per-fold derived seeds 42 + fold index). Backbone for all encoders is a 3D ResNet-18 (stem + layer1-4; channels 64-128-256-512). Tumor masks used as Stage-1 targets were generated by an nnU-Net trained on BraTS (Supplementary Table S1); the full hyperparameter configuration is given in Supplementary Table S3.

```
GLOBAL REPRODUCIBILITY SETTINGS
SEED = 42
function set_seed(s):
   random.seed(s); numpy.seed(s)
   torch.manual_seed(s); torch.cuda.manual_seed_all(s)
Backbone (all encoders): 3D ResNet-18 = stem + layer1..layer4 (channels 64->128->256->512)
Input: single-modality ROI volume resized to 128^3, intensity-normalized.

------------------------------------------------------------------------------
STAGE 1 - Domain-adaptive pretraining (tumor-segmentation supervision only)
------------------------------------------------------------------------------
# Train ONE encoder per modality (T1, T1CE, T2) on all available cases that have
# both the image and a tumor mask. No survival outcomes are used at this stage.
# Network: ResNet-18 3D encoder (initialized from Med3D 3D-pretrained weights)
#         + lightweight U-Net-style decoder (skip connections).
# Segmentation target: 4 classes {0=background, 1=NCR, 2=ED, 3=ET};
#         masks generated by nnU-Net trained on BraTS (Supplementary Table S1).

function stage1_pretrain(modality):              # modality in {T1, T1CE, T2}
   set_seed(SEED)
   cases    <- all (cohort, case_id) with image[modality] AND mask
   shuffle(cases); split 85% train / 15% val
   encoder   <- ResNet18_3D initialized from Med3D pretrained weights
   model    <- encoder + SegDecoder(num_classes=4)
   criterion <- DiceCELoss = 1.0 * DiceLoss(foreground classes 1..3)
                      + 1.0 * CrossEntropy(all classes)
   optimizer <- AdamW(lr=1e-3, weight_decay=1e-4)
   scheduler <- CosineAnnealingLR(T_max=100, eta_min=1e-6)
   scaler    <- GradScaler()                    # AMP (float16)
   best_wt_dice <- 0
   for epoch in 1..100:
      model.train()
      for (mri, mask) in train_loader(batch_size=2, shuffle, augment=True):
         with autocast(float16):
            pred <- model(mri); loss <- criterion(pred, mask)
         scaler.scale(loss).backward(); scaler.step(optimizer); scaler.update()
      scheduler.step()
```

```
      val_wt_dice <- mean whole-tumor Dice on val_loader (augment=False)
      if val_wt_dice > best_wt_dice:
         best_wt_dice <- val_wt_dice
         save encoder.state_dict() -> stage1_encoder_<modality>_best.pth
   return W_enc[modality]                       # reused as Stage-2 initialization
# Stage-1 augmentation (train only): random axis flip; rotation (+/-15 deg);
#   intensity shift (+/-10%).

-----------------------------------------------------------------------------
CORE LOSS - Cox negative log partial likelihood (Breslow)
-----------------------------------------------------------------------------
function cox_loss(risk, time, event):        # risk higher = worse prognosis
   order       <- argsort(time, descending=True)
   risk, event  <- risk[order], event[order]
   log_risk_set <- logcumsumexp(risk)        # log SUM_{j: time_j >= time_i} exp(risk_j)
   if sum(event) == 0:
      return 0 (kept differentiable)
   nll        <- -mean( (risk - log_risk_set)[event == 1] )   # uncensored only
   return nll + 0.01 * mean(risk^2)          # L2 penalty on risk scores

-----------------------------------------------------------------------------
MODEL ASSEMBLY (Stage 2)
-----------------------------------------------------------------------------
function build_model(type):
   if type in {single_T1, single_T1CE, single_T2}:
      m <- SingleModality(encoder); load W_enc[modality] into m.encoder
   elif type == early_fusion:
      m <- EarlyFusion(1 encoder, 3-channel stem); init fused stem from W_enc mean
   elif type == late_fusion:
      m <- LateFusion(3 encoders + feature concat); load W_enc[T1,T1CE,T2]
   elif type == cross_attention:                   # CAF (proposed)
      m <- CrossAttentionFusion(3 encoders + cross-attn, n_heads=4)
      load W_enc[T1,T1CE,T2]
   return m

function freeze_and_group(m, base_lr=1e-4):
   freeze (requires_grad=False): stem, layer1, layer2, layer3
   trainable: layer4 + fusion module + prediction head
   param_groups = [ {layer4 params, lr = base_lr*0.5 = 5e-5},
                 {head/fusion params, lr = base_lr = 1e-4} ]

-----------------------------------------------------------------------------
STAGE 2 - Survival fine-tuning with stratified 5-fold cross-validation
-----------------------------------------------------------------------------
```

```
function stage2_cv(type, records):    # records = 386 patients (Local+CGGA+TCGA)
   set_seed(SEED)
   strat_label <- OS_Status x Cohort          # joint stratification key
   folds <- StratifiedKFold(n_splits=5, shuffle=True, random_state=SEED)
   oof_predictions <- []
   for fold_idx, (train_idx, val_idx) in folds.split(records, strat_label):
      set_seed(SEED + fold_idx)             # per-fold derived seed
      model    <- build_model(type); freeze_and_group(model)
      optimizer <- AdamW(param_groups, weight_decay=1e-3)
      scheduler <- WarmupCosine(warmup=10, total=100, floor=1% of base_lr)
      scaler   <- GradScaler()             # AMP (float16)
      best_ci <- 0; patience <- 0
      for epoch in 1..100:
         model.train()
         for batch in train_loader(batch_size=4, shuffle, augment=True):
            with autocast(float16):
               risk <- model(inputs)
               loss <- cox_loss(risk, time, event)
            scaler.scale(loss).backward()
            clip_grad_norm_(model.params, max_norm=1.0)
            scaler.step(optimizer); scaler.update()
         scheduler.step(epoch)
         val_ci <- concordance_index(model on val_loader)
         if val_ci > best_ci:
            best_ci <- val_ci; best_state <- copy(model.state); patience <- 0
         else:
            patience <- patience + 1
            if patience >= 30: break        # early stopping
      model.load(best_state)
      risk_val <- model(val_loader)           # held-out fold predictions
      append (fold_idx, cohort, case_id, risk_val, os_months, os_status)
           to oof_predictions
   # Out-of-fold predictions = the basis of ALL reported metrics
   save oof_predictions -> stage2_cv_predictions_<type>.csv
   return oof_predictions
# Stage-2 augmentation (train only): random flip, rotation (+/-15 deg), intensity shift
(+/-10%)

------------------------------------------------------------------------------
STAGE 3 - Clinical Cox integration (lifelines)
------------------------------------------------------------------------------
function stage3_cox(oof_predictions, clinical):
   dl_risk_std <- per-fold z-score(risk_score)        # standardized within fold
   features    <- [Age_std, Gender, MGMT_Status, MGMT_missing,
```

```
                Adjuvant_Therapy, dl_risk_std]
   model <- lifelines.CoxPHFitter(penalizer=0.1).fit(features, time, event)
   return hazard_ratios, C-index

----------------------------------------------------------------------------
METRICS (computed on pooled out-of-fold predictions)
----------------------------------------------------------------------------
Harrell C-index; time-dependent AUC (12/18/24 mo); calibration (O/E, slope, CITL);
decision-curve analysis; Integrated Brier Score.
95% CIs: 1000-iteration patient-level percentile bootstrap (fixed seed).
```
